# Transcript-targeted analysis reveals isoform alterations and double-hop fusions in breast cancer

**DOI:** 10.1038/s42003-021-02833-4

**Published:** 2021-11-22

**Authors:** Shinichi Namba, Toshihide Ueno, Shinya Kojima, Kenya Kobayashi, Katsushige Kawase, Yosuke Tanaka, Satoshi Inoue, Fumishi Kishigami, Shusuke Kawashima, Noriko Maeda, Tomoko Ogawa, Shoichi Hazama, Yosuke Togashi, Mizuo Ando, Yuichi Shiraishi, Hiroyuki Mano, Masahito Kawazu

**Affiliations:** 1 Division of Cellular Signaling, National Cancer Center Research Institute, Tokyo, 104-0045 Japan; 2 Department of Head and Neck Oncology, National Cancer Center Hospital, Tokyo, 104-0045 Japan; 3 Department of Gastroenterological, Breast and Endocrine Surgery, Yamaguchi University Graduate School of Medicine, Yamaguchi, 755-8505 Japan; 4 Department of Breast Surgery, Mie University Hospital, Mie, 514-8507 Japan; 5 Department of Translational Research and Developmental Therapeutics against Cancer, Yamaguchi University Graduate School of Medicine, Yamaguchi, 755-8505 Japan; 6 Department of Otolaryngology, Head and Neck Surgery, The University of Tokyo Hospital, Tokyo, 113-8654 Japan; 7 Division of Genome Analysis Platform Development, National Cancer Center Research Institute, Tokyo, 104-0045 Japan; 8 Present Address: Department of Statistical Genetics, Osaka University Graduate School of Medicine, Osaka, 565-0871 Japan; 9 Present Address: Division of Cell Therapy, Chiba Cancer Center, Research Institute, Chiba, 260-8717 Japan

**Keywords:** Breast cancer, Cancer genomics, RNA sequencing

## Abstract

Although transcriptome alteration is an essential driver of carcinogenesis, the effects of chromosomal structural alterations on the cancer transcriptome are not yet fully understood. Short-read transcript sequencing has prevented researchers from directly exploring full-length transcripts, forcing them to focus on individual splice sites. Here, we develop a pipeline for Multi-Sample long-read Transcriptome Assembly (MuSTA), which enables construction of a transcriptome from long-read sequence data. Using the constructed transcriptome as a reference, we analyze RNA extracted from 22 clinical breast cancer specimens. We identify a comprehensive set of subtype-specific and differentially used isoforms, which extended our knowledge of isoform regulation to unannotated isoforms including a short form *TNS3*. We also find that the exon–intron structure of fusion transcripts depends on their genomic context, and we identify double-hop fusion transcripts that are transcribed from complex structural rearrangements. For example, a double-hop fusion results in aberrant expression of an endogenous retroviral gene, *ERVFRD-1*, which is normally expressed exclusively in placenta and is thought to protect fetus from maternal rejection; expression is elevated in several TCGA samples with *ERVFRD-1* fusions. Our analyses provide direct evidence that full-length transcript sequencing of clinical samples can add to our understanding of cancer biology and genomics in general.

## Abstract (web summary)

Namba et al develop a new pipeline called MuSTA to enable the efficient assembly of transcriptome from long-read sequencing data of breast cancer samples. This method enables the authors to discover subtype-specific isoforms, find that fusion transcript structures depend on their genomic context and identify a double-hop fusion that results in aberrant expression of an endogenous retroviral gene.

## Introduction

The transcriptome is an important determinant of cellular phenotype^1^, and changes in the transcriptome are major drivers of oncogenesis and DNA alteration^2^. In some cases, aberrant splicing regulation is recurrent^3^ and considered as a driver independent of somatic mutations^4^. Some genes have cancer-specific splicing isoforms that underlie phenomena related to cancer proliferation, e.g., *PKM2* in the Warburg effect^5^, long non-coding RNA *PNUTS* in the epithelial–mesenchymal transition^6^, and *BRAF* exons 3–9 in chemo-resistance^7^. Because aberrant splicing is one of the hallmarks of cancer, understanding this phenomenon is indispensable for a better understanding of tumorigenesis.

Several groups recently conducted comprehensive studies of cancer-specific alternative splicing^2,8–10^ and showed that RNA alteration affects cancer genes in a manner that complements DNA alteration^2^. However, all of these studies depended on RNA-seq technology, which produces relatively short reads and requires imputation to generate full-length transcripts. Consequently, these analyses were limited to individual splice site abnormalities and could neither directly nor efficiently target consequent transcripts. It is especially difficult to quantify gene expression at the transcript level, and annotation lists based on incomplete sets of isoforms have low estimation accuracy^11^. Transcript expression exhibits a cell type-specific pattern^12^, and far more isoforms exist than are registered in the reference annotation^13^. Therefore, unless we use a complete catalog of transcripts in target cells, it is difficult to correctly evaluate transcript usage.

Complex structural variations (SVs) have been reported in a wide range of cancer types^2^, but because the scale of SVs is far longer than the length of RNA-seq read fragments, only limited aspects of RNA-seq have been captured through previous analyses. Specifically, in triple-negative breast cancer (TNBC), a distinct subtype of breast cancer, the genome is heavily affected by SVs due to a deficiency in homologous recombination^14–16^. However, the characteristics of transcripts derived from genomic regions affected by complex SVs remain to be analyzed.

In this study, we used single-molecule real-time (SMRT) sequencing technology^17^ to sequence breast cancer clinical specimens in order to directly and comprehensively investigate transcript regulation. SMRT sequencing can obtain far longer reads (≥20 kbp) than short-read sequencing, making it possible to read full-length transcripts without fragmentation (IsoSeq protocol)^18^. Several groups have used this approach to capture high-resolution transcriptomes of eukaryotes^19–21^ including human^13^, many of which revealed transcriptome diversity and previously undescribed transcript regulation^22–24^. However, this sequencing method has been applied to only a few individual samples. Furthermore, few studies have used it for cancer, especially for clinical cancer specimens^25,26^. In this study, we constructed a cohort-wide breast cancer transcriptome from directly sequenced transcripts and characterized its complexity and subtype-specific regulation; hundreds of thousands of the isoforms we identified were previously unannotated. We also detected a functional unannotated isoform of *TNS3* that was differentially regulated among subtypes. Furthermore, we examined relationships between the exon–intron structure of fusion transcripts and their genomic contexts, and found functional double-hop fusion transcripts transcribed from three distinct genomic regions involved in complex structural alterations. Our findings show that transcript-targeted analyses can directly capture a catalog of cancer isoforms originating from complex structural alterations.

## Results

### Cohort-wide transcriptome enables more accurate inference of transcript usage

**Fig. 1:**
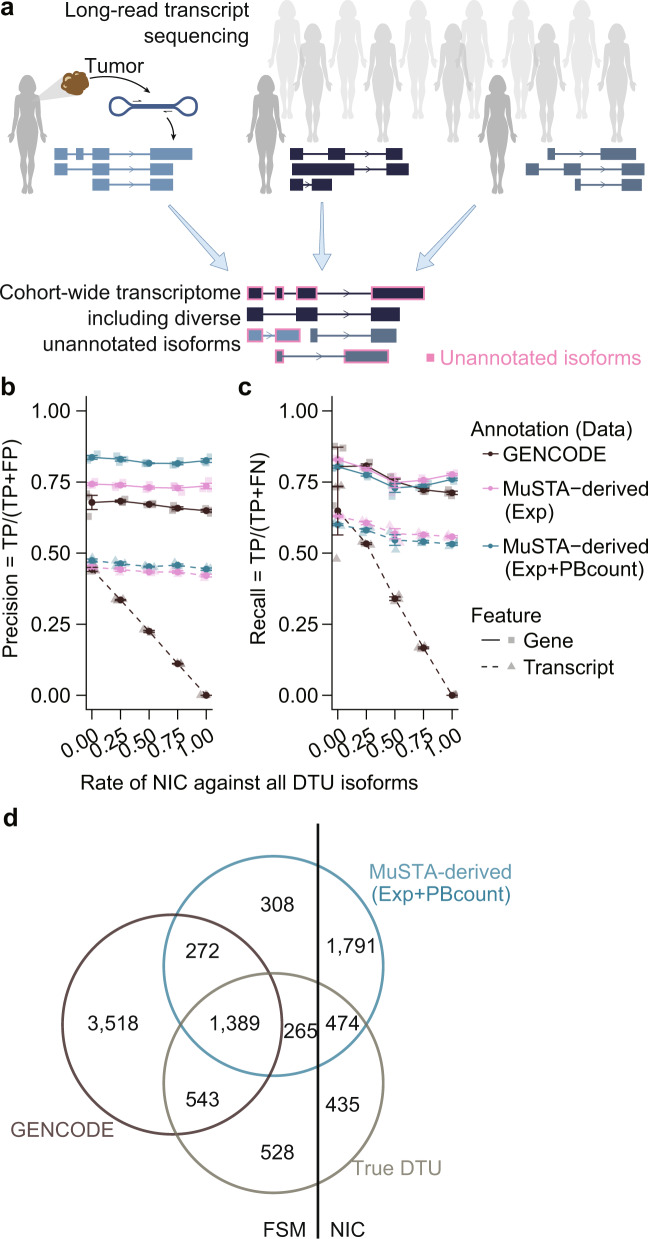
A cohort-wide transcriptome enables more accurate inference of transcript usage. **a** Schematic view of the MuSTA workflow. A detailed scheme is depicted in Supplementary Fig. 1. **b**, **c** We conducted a simulation based on the full-spliced match (FSM) and novel-in-catalog (NIC) isoforms in the breast cancer data set. As originally described in ref. ^27^, FSM isoforms are isoforms for which the splice junctions completely match known isoforms, whereas NIC isoforms contain at least one novel splicing junctions but consist of known splicing donors and acceptors. We permutated the log-averaged expression of FSM isoforms and NIC isoforms separately, and randomly set differential gene expression (DGE) and differential transcript usage (DTU). We tested five conditions of NIC ratio against all DTU isoforms (0, 0.25, 0.5, 0.75, and 1). Even with a value of 0 (i.e., all DTU transcripts were FSM), we observed higher precision (**b**) and compatible recall (**c**) for DTU inference with the MuSTA-derived annotation in comparison with GENCODE. As the NIC rate increased, the MuSTA-derived annotation increasingly outperformed GENCODE, and also exhibited stable precision and recall. The dots represent means, and the error bars represent the standard error of three independent simulations. **d** Venn diagram of DTU isoforms for a representative simulation with a NIC rate of 0.25. Exp expression, TP true positive, FP false positive, FN false negative.

We constructed a cohort-wide transcriptome by merging long-read sequencing of 22 clinical breast cancer specimens. Because long-read consensus sequences are sometimes redundant and distinguished only by sequencing errors, we combined them by focusing on their genomic structures (Fig. 1a and Supplementary Fig. 1). The transcriptome subsequently went through SQANTI^27^ filtering, and potential artifact transcripts were removed by a random forest algorithm. We also obtained a number of uniquely associated full-length non-chimeric (FLNC) reads (hereafter referred to as PBcount) in each sample; PBcount serves as a complementary measure of isoform expression. We named this procedure Multi-Sample long-read Transcriptome Assembly (MuSTA) and evaluated its performance using simulation (Supplementary Note 1 and Supplementary Fig. 2). We also compared the MuSTA-derived transcriptome with the GENCODE reference transcriptome using differential transcript usage (DTU) as the evaluation index, and showed that the former was robust and outperformed the latter in the presence of unannotated isoforms (Fig. 1b–d). DTU is analogous to differential gene expression (DGE), which tells us the variability in the proportion of isoforms at the transcript level. Although current inference methodologies perform poorly in DTU, our results indicated that this might be due to inaccurate annotation at the transcript level, suggesting that direct sequencing of full-length transcriptome could help overcome this limitation and enable us to evaluate transcript usage, even for unannotated isoforms. Comparison of MuSTA with two other pipelines, ToFU^18^ and FLAIR^28^, confirmed that MuSTA can accurately construct transcriptomes with less redundancy (Supplementary Note 2 and Supplementary Figs. 3–6).

### Cohort-wide transcriptome of 22 breast cancer clinical specimens

We sequenced RNA samples from eight estrogen receptor (ER)-positive breast cancer and fourteen TNBC clinical specimens, obtaining a total of 6.15 million consensus reads (median 263,378 reads per sample, Supplementary Fig. 7); these were combined into 818,620 non-redundant isoforms. There were 344,504 isoforms that passed SQANTI (hereafter, MuSTA-transcriptome), including 263,711 (76.5%) unannotated isoforms. The median length of the isoforms was 2,936 nt (interquartile range was 2,196). Among them, 344,429 isoforms were mapped to autosomes or chromosome X; of those, 288,674 (83.8%) had multiple exons. The number of isoforms that passed SQANTI in each sample was between 29,246 and 58,756 (median 39,313) (Supplementary Data 1).

We identified 3,081 unannotated multi-exonic genes. Most were detected only in one sample each, but 41 were detected in multiple samples. Ten genes were unannotated in GENCODE v28, which we used throughout this paper, but newly annotated in GENCODE v34. Furthermore, the MuSTA transcriptome contained 17 translated but unannotated open reading frames (uORFs) identified in ref. ^29^; the authors of that study conducted a CRISPR-based screening to systematically identify ORFs. These results suggested that the MuSTA transcriptome successfully captured isoforms that were actually present but unannotated. In addition, because SQANTI does not acknowledge genomic variants, transcripts with splice sites created by mutation might be deleted as artifacts. Because we could find only seven such isoforms across all the samples, we considered this problem as having a limited effect (Supplementary Data 2).

**Fig. 2:**
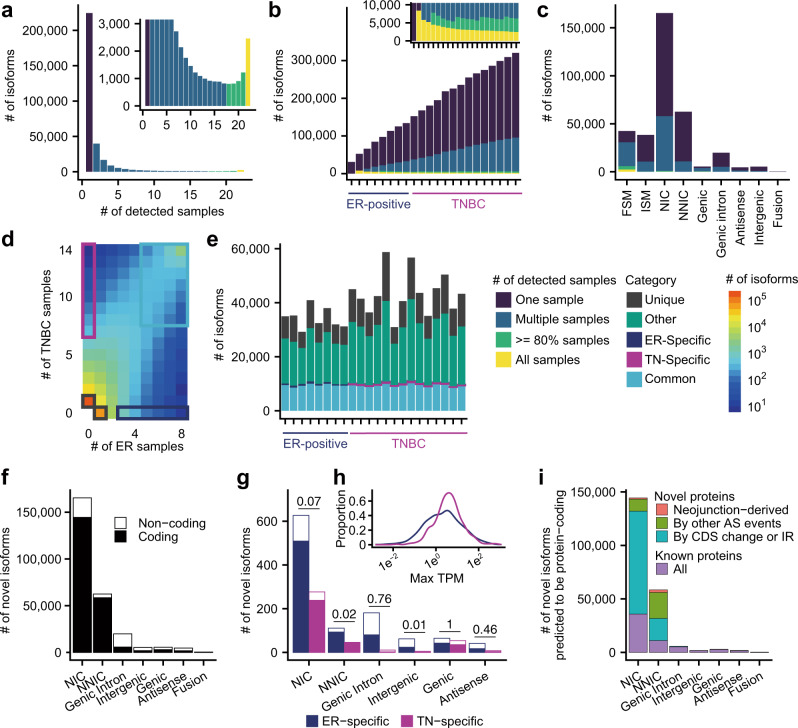
Isoform distribution detected by MuSTA. **a** A histogram of the number of isoforms as a function of the number of samples that generated the isoforms. **b** The number of detected isoforms when incrementing sample numbers one by one and performing MuSTA. **c** The number of isoforms for each SQANTI category, which represents the similarity to reference transcripts. FSM full-splice match, ISM incomplete-splice match, NIC novel in catalog, NNIC novel not in catalog. **d** A heatmap representing the distribution of the number of isoforms from ER-positive and triple-negative subtypes. The groups of isoforms indicated by light blue, blue, purple, and black rectangles were defined as common, ER-specific, TN-specific, and unique, respectively. **e** The distribution of isoforms according to their categories defined in **d**. **f** The number of isoforms restricted to unannotated SQANTI categories. Colored bars represent isoforms predicted to have protein-coding potential. **g** Bar plot representing the number of subtype-specific isoforms. *P*-values for the difference in the ratio of protein-coding isoforms are shown (two-sided Fisher’s exact test, Benjamini–Hochberg corrected). **h** The distribution of maximum transcript per million (TPM) for subtype-specific isoforms. The *x* axis is shown on a logarithmic scale. **i** The origins of novel protein sequences predicted to be transcribed from unannotated transcripts. AS alternative splicing, CDS coding sequence, IR intron retention.

We observed strong heterogeneity of detected transcripts between samples even within the same subtype, with more than half of the isoforms detected only in one sample (Fig. 2a). Furthermore, the number of detected isoforms decreased as the number of samples sharing the isoforms increased (up to 19 samples), indicating that the majority of isoforms were not ubiquitous. Conversely, when the number of samples sharing the isoforms exceeded 19, the number of isoforms increased (Fig. 2a), suggesting that these isoforms are ubiquitous and essential house-keeping transcripts. Next, to determine whether we had used a sufficient number of samples, we increased the number of analyzed samples one by one and applied MuSTA (Fig. 2b). Although the graph did not reach a plateau, we detected a consistent number of isoforms in more than 80% of samples, indicating that we were able to successfully detect most essential transcripts. However, a larger cohort will be required for the thorough investigation of transcriptome heterogeneity. These data indicated a biphasic distribution of isoforms, with strong heterogeneity of minor isoforms and ubiquitous essential transcripts.

We further investigated this heterogeneity with the aid of SQANTI, which classifies isoforms into nine categories by comparison with reference gene annotation: full-splice match (FSM), incomplete-splice match (ISM), novel in catalog (NIC), novel not in catalog (NNIC), genic, genic intron, antisense, intergenic, and fusion. The FSM and ISM categories only contained known splicing junctions. NNIC consisted of isoforms with novel splicing donors or acceptors. NIC isoforms were most abundant, and the pairing of splicing donors and acceptors was much more diverse than what we could find in GENCODE. The second most abundant category was NNIC. Although 80% of those NNIC were detected in only one sample, a certain number of isoforms were recurrently detected. A total of 2765 isoforms were found in all samples; most of these were classified as FSM. On the contrary, almost all isoforms that were classified as genic intron, antisense, intergenic, and fusion were detected only in one sample (Fig. 2c).

Alternatively, we classified the isoforms into four categories from a different point of view: those found in more than half of the samples in both subtypes were defined as “common”; those detected in only one sample were defined as “unique”; isoforms were defined as “subtype-specific” if they were found in only one subtype and the number of detected samples was significantly different between subtypes (*P* < 0.05) in the two-tailed Fisher’s exact test (i.e., more than three samples in ER-positive breast cancer, and more than seven samples in TNBC); and “other” (Fig. 2d). Although the number of unique isoforms varied according to the total number of isoforms in each sample, there was little variation in the number of common isoforms (Fig. 2e). In each sample, 100–200 isoforms were classified as subtype-specific (Supplementary Data 1).

### Repetitive elements in unannotated isoforms

We investigated the repetitive elements in the unannotated isoforms, especially in the intergenic transcripts (Supplementary Notes 3–5 and Supplementary Figs. 8–11). In brief, we took advantage of long-read lengths to successfully map reads containing repetitive sequences using long-read aligners. We detected a substantial proportion of long interspersed nuclear elements (LINEs), long terminal repeats (LTRs), and short interspersed nuclear elements (SINEs) in intergenic transcripts (Supplementary Figs. 8–10). Intergenic genes were co-expressed with their neighbor genes regardless of the contents of repetitive sequences (Supplementary Fig. 11), suggesting that genes originating from repetitive sequences are also involved in *cis*-regulation by local genome architecture. We also identified 154 intergenic genes predicted to encode proteins longer than 50 aa, of which 86 were predicted to localize in the nucleus (Supplementary Note 4).

### Unannotated isoforms as a rich resource of cancer-specific neo-junctions

We also evaluated the protein-coding potential of unannotated isoforms (Fig. 2f). NIC isoforms included large numbers of predicted protein-coding isoforms, but surprisingly, the NNIC isoforms had the largest proportion of isoforms with protein-coding potential. This may be explained by the fact that the NIC isoforms included unspliced (or intron retention) isoforms, which might contain premature stop codons. The protein-coding potential was higher in TN-specific isoforms than ER-specific isoforms in the NNIC and intergenic categories (Fig. 2g). The caveat is that the TN-specific isoforms tended to be expressed at higher levels, as the larger number of TNBC samples resulted in more stringent criteria for subtype specificity (Fig. 2h), potentially confounding the protein-coding potential of the subtype-specific isoforms.

We noticed that a substantial fraction of predicted protein sequences encoded by unannotated isoforms were not registered in databases (Fig. 2i). These isoforms were 2.15 times more abundant in NNIC than in NIC, despite the smaller number of isoforms with protein-coding potential in NNIC. The NNIC transcripts, by definition, had at least one novel splice junction, and therefore tended to encode novel protein sequences. Importantly, 4.1% of these peptide sequences were derived from “neo-junctions”, which were reported as tumor-specific splice junctions in The Cancer Genome Atlas (TCGA) cohort; these sequences are thought to bind MHC-I and act as neo-antigens^10^.

Thus, we identified a large number of potential protein-coding sequences that had not been previously recorded, including a substantial number of alternative splicing events that may produce neo-antigens.

### Subtype-specific isoforms reflect relationships between cancer genes and subtypes

**Fig. 3:**
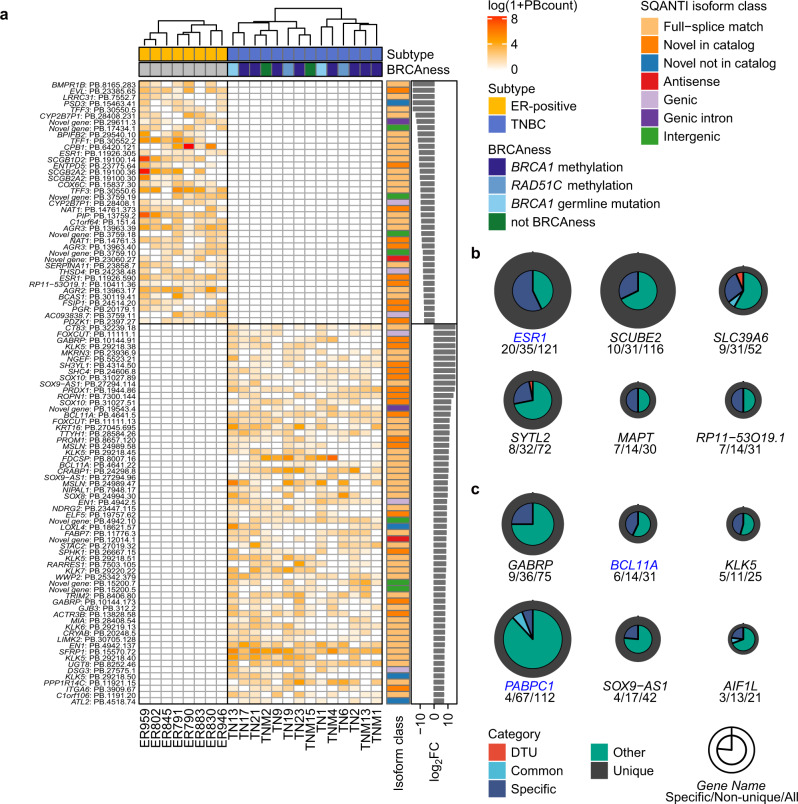
Subtype-specific isoforms reflect relationships between cancer genes and subtypes. **a** The top 100 subtype-specific isoforms with the highest fold changes in transcript per million (TPM). Log-transformed PBcount data in each sample are indicated in the heatmap. Subtype and BRCAness are annotated for samples (top), and isoform classification by SQANTI and log_2_ TPM fold change are annotated for isoforms (right). Absolute values of log_2_ fold change greater than 10 were truncated. **b**, **c** Circular plots indicating the number of isoforms according to isoform categories defined in Fig. 2d and differential transcript usage (DTU). **b** ER-specific. **c** TN-specific. Gene symbols of oncogenes are colored in blue. The three numbers below each gene symbol indicate the numbers of specific (left), non-unique (center), and all (right) isoforms.

We hypothesized that subtype-specific isoforms encode key molecules involved in cellular pathways specific to the corresponding subtypes. To address this, we selected the top 100 subtype-specific isoforms with the highest fold change in transcripts per million (TPM) (Fig. 3a). Isoforms from key oncogenes in the ER-positive subtype, such as the *ESR1* and *PGR* isoforms, were present in the top 100 isoforms. Figure 3a shows NIC isoforms from subtype-specific genes, including an *AGR3* isoform in ER-positive breast cancer^30^ and a *GABRP* isoform in TNBC^31^. Among the NNIC isoforms, *KLK5* is a tumor suppressor gene (TSG)^32^ and *LOXL4* is involved in breast cancer metastasis^33^; moreover, four isoforms of these genes were among the top 100 subtype-specific isoforms. It is likely that the number of subtype-specific isoforms reflected the association of genes with the respective subtypes. In reality, *ESR1* had the largest number of subtype-specific isoforms in ER-positive breast cancer (Fig. 3b). *GABRP* had the largest number of subtype-specific isoforms in TNBC, and other oncogenes such as *BCL11A* and *PABPC1* also had many TNBC-specific isoforms. The results of this analysis may lead to the identification of novel oncogenes associated with breast cancer. For example, 17 isoforms of unknown origin (novel genes) were among the top 100 isoforms, and these warrant further investigation.

To validate the existence of isoforms detected in MuSTA, we focused on *SOX9-AS1*, a long non-coding RNA on the antisense strand of the transcription factor gene *SOX9*. In our data, two isoforms were expressed strongly in TNBC (Fig. 3a), and 42 isoforms including four TNBC-specific isoforms were detected (Fig. 3b). We also detected readthrough transcripts spanning *SOX9-AS1* and its adjacent gene, *AC005152.3*. Using nested PCR, we validated the existence of these isoforms (Supplementary Fig. 12).

### Differential transcript usage analysis with MuSTA-transcriptome captured previously reported isoform switching

**Fig. 4:**
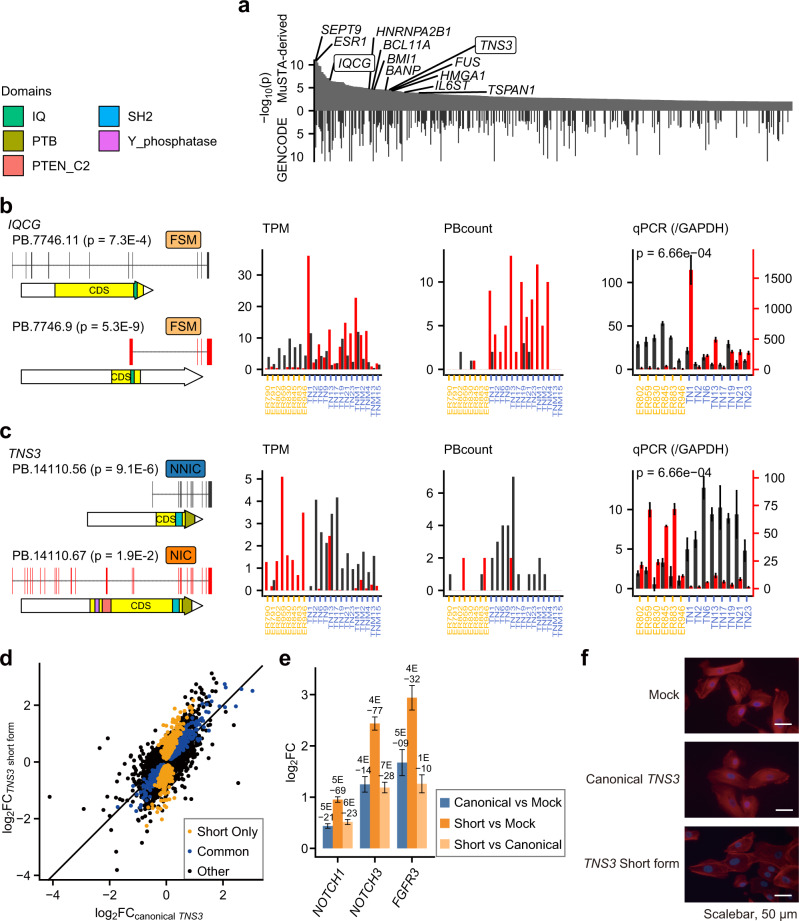
Differential transcript usage in the MuSTA transcriptome. **a** −Log_10_ *P*-value of differential transcript usage (DTU) inference in isoforms with *P* < 0.01 when using the MuSTA transcriptome. *P*-values were corrected in a stage-wise manner described in ref. ^56^. −log_10_ *P*-values for GENCODE annotation are shown for isoforms that are annotated in GENCODE. We sorted genes in the ascending order of DTU *P*-values corresponding to the MuSTA transcriptome. The gene symbols with the smallest *P*-values are labeled according to whether they are oncogenes or TSGs. Two genes were validated by qRT-PCR, and are labeled with boxes. **b**, **c** qRT-PCR validation of *IQCG* (**b**) and *TNS3* (**c**). Shown are SQANTI classifications, transcript structures, predicted protein domains of two DTU isoforms with the smallest *P*-values, and expression of DTU isoforms. Three types of expression data are shown [transcript per million (TPM) aligned to the MuSTA transcriptome, PBcount, and relative qPCR expression against *GAPDH*]. Relative qPCR expression has two *y*-axes along with DTU isoforms, because qPCR was conducted separately for each isoform. Error bars in the qPCR graphs indicate the standard deviation of three replicates. *P*-values for relative expression of DTU isoforms were calculated by a two-tailed Mann–Whitney *U* test. **d** Log_2_ fold changes of gene expression in *TNS3*-expressing MCF10A cells against MCF10A cells transduced with mock vector. The definitions of “short only” and “common” genes are visualized in Supplementary Fig. 14d. **e** Log_2_ fold changes of *NOTCH1*, *NOTCH3*, and *FGFR3* expression between MCF10A cells expressing *TNS3* short form, canonical *TNS3*, and mock vector. Error bars represent the standard error. **f** MCF10A cells, into which viral vectors were introduced, were stained with Alexa Fluor 594-labeled phalloidin to visualize actin organization. Nuclei were counterstained with Hoechst 33258.

As another approach to capture subtype-related isoforms, we conducted DTU tests with the transcriptome obtained by MuSTA, assuming that those genes have functional relevance to breast cancer biology. We detected 465 DTU genes (FDR < 0.01), including 46 cancer-related genes and 10 genes specifically related to breast cancer, including *ESR1* and *BCL11A* (Fig. 4 and Supplementary Figs. 13a–c).

Among DTU genes, *MED24* has canonical and short isoforms; the former is expressed ubiquitously, whereas expression of the latter is specific to a highly metastatic mouse breast cancer cell line (4T1)^34^. We confirmed the ubiquitous expression of the canonical isoform and found that the short isoform was selectively suppressed in TNBC (Supplementary Fig. 14a), but we failed to replicate this finding by PCR. However, the isoform expression in mouse was validated by PCR in the original article. The differences in our PCR results might be due to the difficulty of designing PCR primers for *MED24*, as all of the exons of *MED24* short isoform are present in the long isoform. Given that the 4T1 cell line is a triple-negative cell line, escape from the regulation of short-form *MED24* might be associated with metastasis.

Another DTU gene, *TPD52*, which has a short isoform encoding PrLZ, is a biomarker of prostate cancer and has an anti-apoptotic function^35,36^. This isoform exhibits androgen-dependent expression in prostate cancer^37^. We observed expression of the short isoform specific to ER-positive cancers through our analysis (Supplementary Fig. 14b).

*IQCG* intronic TSS induces the overexpression of exons 9–12, and Bjørklund et al. pointed out that deregulated expression in this region might be oncogenic^38^. Using MuSTA and an additional RT-qPCR experiment, we confirmed that PB.7746.9 (matched to ENST00000478903.5) was overrepresented relative to other isoforms in TNBC, and that this matched the reported intronic-start transcript (Fig. 4b). Based on these findings, we conclude that MuSTA successfully captured known isoform-switching events.

Next, we conducted Gene Ontology and KEGG pathway enrichment analysis for the DGE and DTU genes (Supplementary Fig. 13d, e). In DGE genes, pathways related to peptidase processing, ectodermal development, and the cell cycle were enriched. Curiously, eight out of the top ten biological processes enriched in DTU genes were associated with molecular binding. mRNA metabolic process, cell division, and RNA processing were among the molecular functions enriched in DTU genes. The two significantly enriched KEGG pathways were the spliceosome and the cell cycle. Therefore, isoform switching is implicated in the regulation of the cell cycle and RNA processing. Notably, although DTU genes (and in particular, DTU isoforms) were not identical between the MuSTA and GENCODE transcriptomes, these findings remained true (Supplementary Fig. 13f, g).

### The short form of *TNS3* has a different function from canonical *TNS3*

Given the recapitulation of known isoform switching by MuSTA, we focused on a DTU event of *TNS3* (Tensin3), which included an unannotated short NNIC isoform (PB.14110.56) (Fig. 4c). Tensin3, a protein with an SH2 domain and a C2 domain, contributes to cell migration, anchorage-independent growth, and metastasis in several types of cancer, including breast cancer^39–41^. Although the functional differences in *TNS3* isoforms have not been extensively studied, several single-nucleotide polymorphisms in 7p12.3 have been reported as pleiotropic cancer susceptibility loci^42^ and coincide with splicing quantitative trait loci (sQTLs) of *TNS3*^43^, suggesting that alternative transcription of *TNS3* plays an important role in cancer.

The *TNS3* short form had an unannotated first exon whose genomic sequence is conserved among vertebrates. In addition, this first exon was associated with peaks of chromatin modifications associated with promoter or enhancer elements in TNBC cell lines and normal breast epithelial cells, whereas these peaks were not observed in ER-positive cell lines (Supplementary Fig. 14c). These findings support the existence of the unannotated isoform and suggest that it is under epigenetic control.

To investigate the function of the *TNS3* short form, we conducted RNA-seq and analyzed the gene expression profiles of MCF10A immortalized mammary epithelial cells into which viral vectors expressing canonical *TNS3* or the *TNS3* short form had been introduced. DGE analysis revealed that the *TNS3* short form had an impact on gene expression similar to that of canonical *TNS3*, but with larger effect sizes (Fig. 4d, e and Supplementary Data 3). In addition, more genes (881) were affected specifically by the *TNS3* short form (Supplementary Fig. 14d). Gene Ontology analysis suggested that *TNS3* altered several features related to cellular adhesion, and that the *TNS3* short form had a larger impact than canonical *TNS3* (Supplementary Fig. 14e, f). “Regulation of extracellular matrix” was one of the gene ontologies enriched in cells expressing short-form *TNS3*, suggesting its association with metastasis. The formation of actin filament stress fibers was indeed enhanced in cells expressing the *TNS3* short form (Fig. 4f). We also found that expression of *NOTCH1*, *NOTCH3*, and *FGFR3*, important drivers of TNBC, was higher in MCF10A cells expressing the *TNS3* short form (Fig. 4e). Together, these data showed that the *TNS3* short form has a function distinct from that of canonical *TNS3*, and may drive tumor initiation or progression. We also noticed that expression of the *TNS3* short form, but not that of canonical *TNS3*, in MCF10A cells was induced by TGFβ (Supplementary Fig. 14e). These observations strongly indicated that isoform switching of *TNS3* has important functional implication for cancer cell phenotype, although further investigation is required to delineate the functional significance and mechanistic basis.

Next, we verified the expression of the *TNS3* short form in TCGA by calculating percent-spliced-in (PSI) of its first intron. We confirmed that PSI was higher in basal breast cancer than luminal A or luminal B breast cancer; surprisingly, high PSI was also observed in a broad range of cancer types, specifically in glioblastoma multiforme, brain lower-grade glioma, and chromophobe renal cell carcinoma (Supplementary Fig. 15). To examine the association of the *TNS3* short form with prognosis, we conducted regression analyses under Cox proportional hazards models (Supplementary Data 4 and “Methods” section). Higher PSI was associated with significantly worse prognosis for OS, DSS, and DFI in kidney renal papillary cell carcinoma (Benjamini–Hochberg adjusted FDR < 0.05) and for OS and DSS in stomach adenocarcinoma. Nominal significance (unadjusted *P* < 0.05) was observed for OS of head and neck squamous cell carcinoma; DSS of lung adenocarcinoma; DFI of lung squamous cell carcinoma; and PFI of glioblastoma multiforme, stomach adenocarcinoma, bladder urothelial carcinoma, and chromophobe renal cell carcinoma. On the other hand, the association was not significant for breast cancer. This might be because the effect of TNS3 was canceled out by the dependence of the *TNS3* isoform switch on the breast cancer subtype. Therefore, we restricted the analysis to the basal subtype and observed a hazard ratio greater than one, although it was not significant, probably due to the small sample size (*n* = 148). Overall, these data indicated that the *TNS3* short isoform was associated with poor prognosis in a wide range of cancer types, although confirmation of this association will require additional investigation in a larger cohort.

### Structure of fusion transcripts reflects the genomic context

Although short-read sequencing makes it possible to detect the breakpoints of structural variation with high sensitivity and accuracy, long-read sequencing enables us to see the structure of resultant transcripts accurately to an extent that could not be achieved with short-read sequencing.

**Fig. 5:**
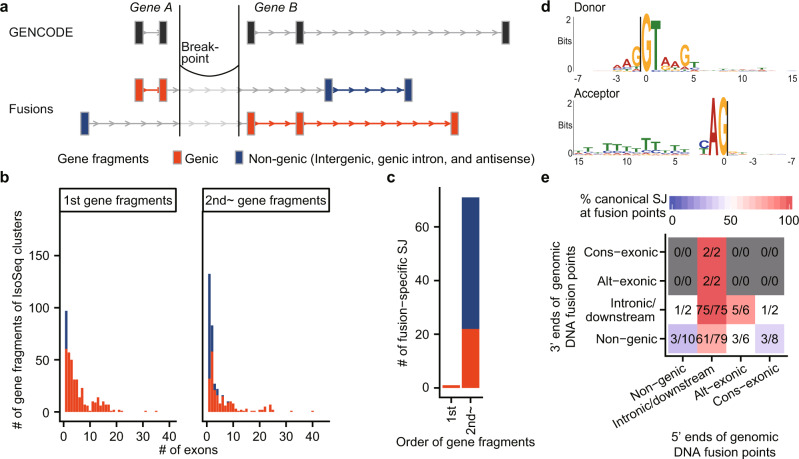
Transcript structure of fusion transcripts. **a** Schematic of gene fragments. **b** Distribution of the number of exons per gene fragments according to their genomic regions and order in fusion transcripts. **c** Bar plots showing the number of fusion-specific splicing junctions for each order of gene fragments in fusion transcripts. **d** Motif of fusion-specific splicing junctions. Note that the first two intronic bases were intentionally chosen. **e** A heatmap with axes representing exon–intron structures at both edges of fusion points. Colors and numbers indicate the proportions of confirmed canonical splice sites at fusion points. “Non-genic” means that the corresponding gene fragments were mapped to an intergenic, genic intron, or antisense regions. “Intronic/downstream” means that a fusion point is on an intron or downstream of genes. “Alt-exonic” means an alternative exon, and “cons-exonic” means a constitutive exon.

Of the chimeric IsoSeq cluster reads found in nine TNBC samples, we identified 402 reads with corresponding breakpoints in whole-genome sequencing (WGS) data (Supplementary Fig. 16a). When the transcript fragments 5′ and 3′ of the fusion points were multi-exonic, almost all were mapped to genic regions. By contrast, when they were mono-exonic, more than half were mapped onto non-genic regions (intergenic, genic intron, or antisense regions) (Fig. 5a, b). Almost all transcript fragments containing TSSs were mapped to genic regions, versus only half of the downstream fragments, suggesting that transcriptional initiation has a stringent requirement for a known TSS.

Next, in order to characterize the aberrant transcription caused by chromosomal rearrangement, we examined fusion-specific splicing junctions. We defined fusion-specific splicing junctions as splicing junctions that exist on neither GENCODE transcripts nor non-chimeric MuSTA isoforms. Most of them were located 3′ of the fusion points (Fig. 5c). A total of 49 (68%) fusion-specific splice junctions were found in non-genic regions, whereas others were detected in genic regions. The motif of fusion-specific splicing junctions was similar to that of ordinary canonical junctions (Fig. 5d), with the caveat that the first two intronic bases were intentionally chosen because we removed chimeric reads with non-canonical junctions (other than GT-AG, GC-AG, and AT-AC) that were not detected in the GENCODE or MuSTA transcriptome.

Next, we examined the genomic DNA sequences of fusion points in association with the fusion-specific splice junction. When we sorted reads according to genomic contexts of fusion points (Fig. 5e), the 5′ ends of fusion points were found mostly within introns or downstream of the genes to which the fusion transcript fragments were assigned. When both sides of genomic DNA fusion points were located at introns or downstream of genes, transcribed and spliced sequences matched the canonical splicing motif in all cases (75/75), indicating that novel exons or splice junctions concordant with splicing rules were indeed generated in association with chromosomal rearrangements. In addition, there were a few reads that matched the splicing motif even when the 5′ ends or 3′ ends of the fusion points were on constitutive exons. Thus, it is reasonable to speculate that the exon–intron structures in these reads changed based on structural context.

### Double-hop fusion transcripts originated from complex genomic structural variations

**Fig. 6:**
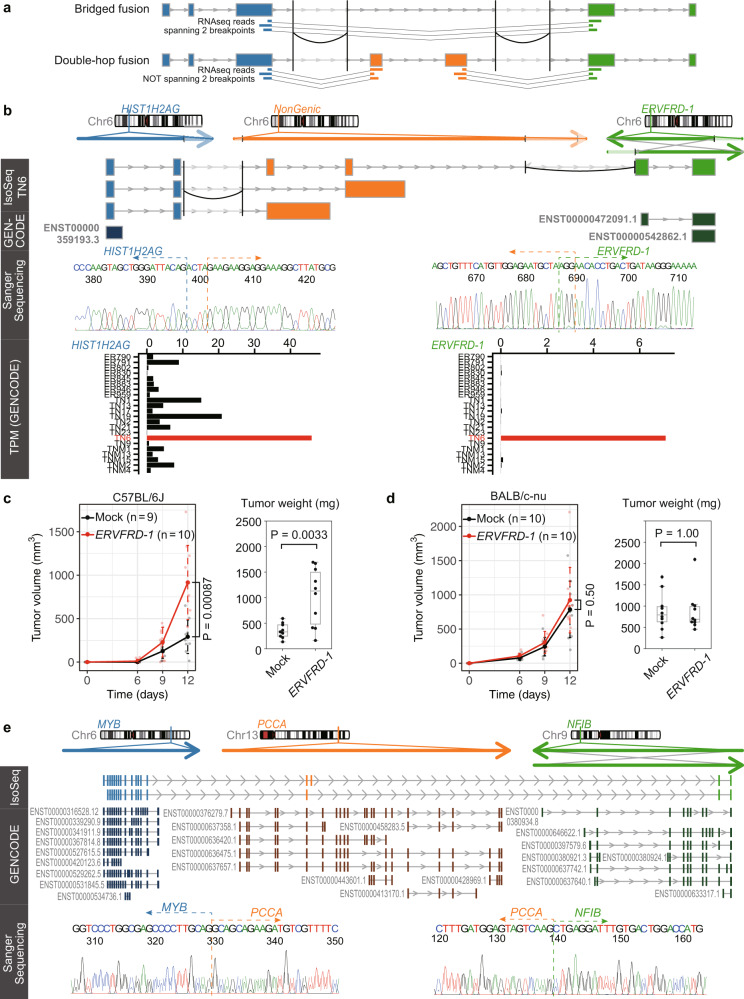
Double-hop fusion. **a** Schematic image of difference between bridged fusion transcript and double-hop fusion transcript. **b** Structure of double-hop fusion transcripts from *HIST1H2AG–NonGenic–ERVFRD-1*. The genomic axes represent three original genomic regions; below them are chimeric IsoSeq cluster reads. Curves correspond to structural variants detected with whole-genome sequencing data. The category “GENCODE” shows annotated transcripts. Outside regions of structural variants are shaded. To ensure visibility, exon–intron structures do not necessarily reflect accurate length. TPM denotes transcript per million. **c**, **d** Growth of MC38 tumor cells expressing *ERVFRD-1* in C57BL/6J mice (**c**) and in BALBc/nu-nu mice (**d**). Error bars represent standard error. **e** Structure of double-hop fusion transcripts from *MYB–PCCA–NFIB*. Representative transcripts are shown for the GENCODE transcriptome.

The concordance of splicing rules in fusion transcripts raised further questions. Do complex structural variations (SV) produce transcripts that undergo splicing regulation? Are they functional, possibly even acting as oncogenic drivers? In recent years, long-read genomic sequencing has been used to identify complex SVs that were impossible to detect with next-generation sequencing^44,45^; however, these questions are yet to be answered. Of the chimeric IsoSeq reads, we have identified five non-redundant reads that were mapped to three regions and had breakpoints that were detected in WGS data (Fig. 6a, b and Supplementary Figs. 16b, c, Supplementary Data 5). We have confirmed the fusion transcripts of *HIST1H2AG–NonGenic–ERVFRD-1*, *OGG1–NonGenic–NonGenic*, and *SLC12A2–NonGenic–SLC12A2* by Sanger sequencing of PCR amplicons. Note that two fusion reads, *HIST1H2AG–NonGenic–ERVFRD-1* and *SMIM13–NonGenic–NonGenic*, were transcribed from the sense and antisense strands of the same rearranged locus, although we could not PCR-amplify the latter fusion transcript. Very recently, the Pan-Cancer Analysis of Whole Genomes consortium found several bridged fusion transcripts that mapped to two genomic regions connected by a non-transcribed genomic fragment^2^. However, the double-hop fusions we found had internal genomic regions of thousands of base pairs, and some fusions were even spliced in these regions (Fig. 6a). This type of fusion transcripts cannot be found without long-read transcriptome sequencing.

Because *HIST1H2AG–NonGenic–ERVFRD-1* contains the full CDS of *ERVFRD-1*, full-length ERVFRD-1 protein might be translated from the fusion transcript. *ERVFRD-1* was specifically expressed in samples carrying the fusion transcript. Expression of *ERVFRD-1* is generally suppressed across all tissues, except in the placenta^43^. Furthermore, considering the chromatin modification status, it is quite possible that this double-hop fusion transcript utilized the *cis*-regulatory region of *HIST1H2AG* observed in one normal breast epithelial cell line and two breast cancer cell lines (Supplementary Figs. 17a–c), suggesting the existence of a mechanism similar to enhancer hijacking^46^. The most important difference between this case and enhancer hijacking is that the promoter and enhancer region of *HIST1H2AG* was located 50 kb upstream of *ERVFRD-1* even in a rearranged chromosome, and *HIST1H2AG–NonGenic–ERVFRD-1* used the region by forming the readthrough transcript that spanned two breakpoints.

We also noticed that *ERVFRD-1* was highly expressed in four samples from TCGA that carry *ERVFRD-1* fusions (*ABHD12–ERVFRD-1*, *ELOVL2–ERVFRD-1*, *NEDD9–ERVFRD-1*, and *NOL7–ERVFRD-1*, respectively, according to FusionGBD^47^) (Supplementary Fig. 17d). Because *ERVFRD-1* inhibits antitumor immunity in an allogeneic mouse tumor model^48^, we measured the growth of tumor cells expressing *ERVFRD-1* in a syngeneic mouse model using MC38 murine colon cancer cells derived from a C57BL/6J mouse. MC38 cells expressing *ERVFRD-1* generated larger tumors in C57BL/6J mice than control MC38 cells infected with empty vector (Fig. 6c). By contrast, MC38 cells expressing *ERVFRD-1* and control MC38 cells formed tumors of similar sizes in BALBc/nu-nu mice (Fig. 6d). Similar results were obtained using the murine mammary carcinoma cell line EMT6, although the results were marginally significant (Supplementary Fig. 18). These results indicated that *ERVFRD-1* promoted tumor growth by inhibiting the antitumor immune response of the host mice, and that enhanced expression of *ERVFRD-1* in human cancer cells might contribute to the growth of tumors as well.

The identification of functional double-hop fusion genes prompted us to search for other examples. In a literature search, we noticed that adenoid cystic carcinoma (ACC) occasionally carries cryptic *MYB–NFIB* fusion genes in which an intervening sequence is inserted between the *MYB* and *NFIB* transcript fragments, although the genomic configurations of the cryptic fusion genes were not determined^49^. Coincidentally, we identified double-hop fusion gene *MYB–PCCA–NFIB* among ACC tumors, which we are now investigating in another project independent of this study. To confirm the existence of the fusion gene, the entire coding sequence of the fusion gene was amplified by PCR. By cloning the PCR amplicons, we identified two variants of the fusion genes (Fig. 6e). One of them (the lower variant in the figure) was an in-frame fusion, suggesting that it was a driver fusion event. Although we did not determine the genomic configuration of the fusion gene, it was very likely that the fusion gene was transcribed from three parts of the rearranged genome, as there were at least two splicing variants corresponding to the *PCCA* transcript, and one of the variants was spliced within the *PCCA* locus. Thus, we identified another example of a double-hop fusion gene.

## Discussion

Here, we have described the diversity and heterogeneity of breast cancer transcripts at both the inter-subtype and intra-subtype levels. The transcriptome we determined allowed us to conduct comprehensive analyses of subtype specificity and DTU using isoforms expressed in target sample groups. These analyses recapitulated known transcript-level regulation of cancer-related genes and also revealed an unannotated isoform of *TNS3* as a novel driver of breast cancer.

*TNS3* is known for its contribution to metastasis; its SH2 and C2 domains promote cell migration and metastasis by binding other molecules in the epidermal growth factor receptor (EGFR) signal transduction pathway^39–41^. Epidermal growth factors simultaneously regulate TNS3 and TNS4, also known as C-terminal tensin-like protein (CTEN), to promote mammary cell migration^41^. Although further investigation is required to obtain mechanistic insights, it is clear that the protein encoded by the *TNS3* short form lacks the N-terminal C2 domain and a structure similar to that of CTEN, which might result in functional differences between the *TNS3* short form and canonical *TNS3*.

Furthermore, we used the full-length sequencing capability of IsoSeq to reveal several features of the exon–intron structures of fusion transcripts and fusion-specific splice sites. In addition, we detected double-hop fusion transcripts. Double-hop fusions can be detected by RNA-seq if their central exons are small enough. A few studies have reported sporadic cases^50–52^. Our discovery and PCR validation of multiple double-hop fusions that underwent canonical splicing establishes the concept of double-hop fusions and indicates that they are prevalent in cancer. The putative driver fusion (*MYB–PCCA–NFIB*) and the enhancer hijacking-like mechanism (*HIST1H2AG–NonGenic–ERVFRD-1*) shed new light on the role of complex SVs and splicing in cancer transcriptomics. We believe that further research will reveal unknown functions of double-hop fusions, giving us insights into new mechanisms of tumorigenesis.

To summarize, full-length transcript sequencing in multiple samples provides a transcript-level analysis that complements conventional RNA-seq approaches, enabling us to focus on isoforms from target cells and apply pre-existing analytical methods such as clustering, DGE, DTU, and Gene Ontology analysis. In addition to long-read RNA sequencing data, MuSTA requires reference genomic sequence and transcriptome data as mandatory inputs; these are available for human and several other species. There are two major long-read sequencing techniques, SMRT sequencing from Pacific Biosciences and nanopore sequencing from Oxford Nanopore Technologies (ONT). Although we used SMRT sequencing reads in this paper, MuSTA could theoretically be applied to ONT reads as well.

Our methods have the potential for a variety of uses. In light of the emerging evidence that gene isoforms are responsible for cancer survival^53^ and drug response^54^, elucidating cancer profiles at the isoform level might provide further druggable targets or previously undiscovered biomarkers. In future work, we will investigate cancer-specific isoforms as sources of neo-antigens. Beyond cancer, there is increasing evidence that variant-induced splice alteration leads to a wide range of diseases such as autoimmune and neurological disorders. With a larger cohort, it might be possible to perform QTL analysis at the isoform level; we predict that such an approach would have a more direct impact on the study of gene functions than sQTLs.

Our analysis had several limitations. First, the most important limitation was the lack of sufficient sample numbers to detect isoforms expressed at low levels. Also, because it is difficult to efficiently amplify long (>6 kb) transcripts, long transcripts might be only partially read. Currently, technologies for long-read sequencing are developing rapidly. For example, according to Pacific Biosciences, the newly developed Sequel II generates at least 8-fold more data and far longer raw reads than the conventional Sequel, (https://www.pacb.com/blog/award-winning-sequel-ii-system). The advance of SMRT sequencing will improve the comprehensiveness of isoform detection, and transcript-level expression analysis can be expected to increase in accuracy. Second, although we mainly targeted the isoforms shared across several samples, each sample contained a substantial number of unique isoforms that deserve further evaluation. These isoforms may reflect sample-specific states including somatic mutation and epigenetic alteration^55^, or may simply be the result of aberrant splicing coupled with an elevation of gene expression. Third, our procedure was not fully annotation-free because we used SQANTI filtering, although this approach provides a cohort-wide transcriptome that contains a large number of unannotated isoforms.

Despite these limitations, our findings revealed functional unannotated isoforms that contribute to carcinogenesis. In this report, we have shown that cohort-wide full-length transcriptome sequencing is a unique and useful tool, unveiling complex aspects of gene regulation in the cancer transcriptome that could not be directly evaluated by short-read RNA-seq. Hence, we believe that the approach described here will play an essential role in advancing cancer biology.

## Methods

### Tumor samples

Surgically resected breast tumors were obtained from patients treated at the Yamaguchi University and Mie University Hospital. The patients gave informed consent prior to their participation in the study. The study was approved by the Ethics Committee of the National Cancer Center Research Institute (#2015-202).

### Reference genome and annotation files

We used hg38 as the reference genome and the GENCODE version 28 comprehensive gene annotation as the annotation file, unless otherwise noted. We focused on isoforms mapped to autosome or chromosome X after we applied our pipeline.

### Definition of oncogenes and tumor suppressor genes (TSGs)

We defined oncogenes as those identified in at least one of three curated oncogene databases: Cancer Gene Census^57^ (version 90), ONGene^58^, and OncoKB^59^ (version 1.23). Because ONGene collected only oncogenes, we used the other two repositories for TSGs.

### Whole-genome sequencing (WGS)

WGS was conducted as described previously^15^. The original data are publicly available (https://humandbs.biosciencedbc.jp/en/hum0094-v3#WGS). In this study, we re-analyzed the data as follows. We detected mutations and structural variants (SV) in two ways and combined them together. First, as shown in our previous research^15^, we used in-house pipeline for analysis. Because we worked with hg19 for our pipeline, the results were transferred to hg38 using the liftover tool. Next, to detect short structural variants (SVs), we used Genomon^60^, an analytic pipeline for next-generation sequencing data, which carries out mapping using STAR^61^, annotation, and additional functions including detection of SVs for DNA and intron retention for RNA.

### RNA-seq

Transcriptome sequencing (RNA-seq) was conducted as reported previously^15^. The original data are publicly available (https://humandbs.biosciencedbc.jp/en/hum0094-v3#RNA-seq). RNA-seq was performed with 100 bp paired-end reads using the NEBNext Ultra Directional RNA Library Prep Kit for Illumina (New England BioLabs, Ipswich, MA, USA). In this study, we re-analyzed the data as follows. RNA-seq reads were mapped to the hg38 reference assembly, and expression data were calculated as transcripts per million (TPM) in two ways: by the quasi-mapping-based mode of Salmon^62^ and the STAR-RSEM protocol^61,63^. The Salmon index was used with the ‘–keepDuplicates’ option, and Salmon quant was performed with ‘-l A –gcBias –seqBias –validateMappings’ options. RSEM was performed with the following commands:

- rsem-prepare-reference –gtf –star
- rsem-calculate-expression –star –paired-end <short-1.fastq><short-2.fastq>

We detected splice junctions and intron retention using Genomon, which ran STAR internally for mapping to the reference genome.

For the Annotation file, we used GENCODE version 28 or a set of transcripts retrieved from our SMRT analysis pipeline.

### BRCAness

We defined the triple-negative breast cancers with defective homologous recombination (BRCAness) based on profiles of structural variations, mutational signatures, germline mutational status of BRCA1, expression of BRCA1 and RAD51C, and promoter methylation of *BRCA1* and *RAD51C* as described previously^15^.

### SMRT sequencing

Long-read sequencing was performed using the Pacific Biosciences Single-Molecule Real-Time (SMRT) sequencing technology with SMRT cell chemistry (SMRTbell Template Prep Kit 1.0, Sequel Binding Kit 2.0, Sequel Sequencing Kit 2.0, all from Pacific Biosciences, Menlo Park, CA, USA). Full-length cDNA libraries were constructed from 1 µg of total RNA using the SMARTer cDNA synthesis kit (Takara Bio, Kusatsu, Japan), utilizing the switching mechanism at the 5′ end of RNA template (SMART) technology coupled with PCR amplification. PCR amplification was performed with PrimeSTAR GXL DNA Polymerase (Takara Bio). The sequencing templates used for SMRT sequencing on the Sequel platform (SMRTbell) were constructed from 1 µg of PCR products. After DNA damage and end repair, the SMRTbell adapters were ligated onto the PCR amplicons, followed by purification with 0.6 volumes of Agencourt AMPure PB (Pacific Biosciences) with 10-minute incubation on a vortex mixer. Primer annealing and DNA polymerase binding were carried out according to the manufacturer’s instructions. Briefly, sequencing primers were annealed to the template at a final concentration of 0.833 nM by denaturing the primer at 80 °C for 2 min and cooling to 4 °C before incubation with the library at 20 °C for 30 min. Distributions of SMRTbell size are presented in Supplementary Fig. 7.

This library underwent sequential DNA replication, with DNA polymerase detachment as replication limitation, and was analyzed by IsoSeq2 pipeline using SMRTlink^18^ with the following settings: maximum dropped fraction, 0.8; maximum subread length, 15,000; minimum subread length, 50; minimum number of passes, 1; minimum predicted accuracy, 0.8; minimum read score, 0.65; minimum SNR, 3.75; minimum *Z* score, −9999; minimum quiver, 0.99; trim QVs 3′, 30; trim QVs 5′, 100; minimum sequence length, 200; polish CCS, false; emit individual QVs, false; and required polyA, true. In IsoSeq, consensus reads “Read of Insert (RoI)” were obtained. RoIs with both cDNA primers and poly(A) were defined as full-length (FL) reads, and others were defined as non–full-length reads. IsoSeq clustered these reads into isoform sequences using an algorithm called ICE. “Polished” reads from the algorithm (IsoSeq2 output files polished_hq.fastq and polished_lq.fastq) were subjected to further analyses. Note that those reads do not necessarily represent non-redundant isoforms due to the characteristics of ICE and natural 5′ degradation of RNA.

### Hybrid error correction

We used LoRDEC^64^ for hybrid error correction of IsoSeq reads with RNA-seq data. LoRDEC was executed with following commands:

- lordec-build-SR-graph -T 3 -2 <RNA-seq_interleaved.fastq>-k 19 -s 3 -g
- lordec-correct -T 8 -i <Isoseq_reads.fastq>-k 19 -s 3 -2 <RNA-seq_interleaved.fastq>-o <corrected_Isoseq.fastq>

### Mapping of corrected IsoSeq reads

Next, IsoSeq reads were mapped to the hg38 reference assembly by Minimap2^65^ using two similar commands to retrieve results as both SAM and PAF formats under the same conditions:

- minimap2 -ax splice -uf -C5 –secondary=no <GRCh38.mmi><corrected_Isoseq.fastq>
- minimap2 -cx splice -uf -C5 –cs –secondary=no <GRCh38.mmi><corrected_Isoseq.fastq>

We filtered IsoSeq reads with mapping quality greater than 50.

### Intra-/inter-sample collapsing IsoSeq reads

Intra-sample integration of mapped IsoSeq reads, followed by inter-sample integration, was performed using our R code. For multi-exon transcripts, we merged IsoSeq reads with the same splice junctions. The most upstream TSS and the most downstream TTS of original transcripts of merged isoforms were defined as the TSS and TTS of the merged isoforms, respectively, with all the original TSS and TTS information linked and retained. We combined 5´ truncated multi-exon isoforms with longer and compatible isoforms for each sample separately (meaning, we treated them as fragments of longer transcripts.) On the other hand, in order to detect correct exon–intron structures from transcripts expressed in target cells, we considered those truncated transcripts as independent transcripts of longer transcripts from other samples unless they shared all splice junctions. For single-exon transcripts, we consolidated reads with other single-exon transcripts if the genomic range of the former overlapped with the latter read. We did not combine single-exon transcripts with longer multi-exon transcripts. Both intra-sample and inter-sample integrations were performed according to this procedure.

We considered that an isoform was detected in a particular sample only when there were non-truncated IsoSeq reads in the sample (i.e., multi-exon IsoSeq reads of which all splice junctions matched, or mono-exon IsoSeq reads with genomic range within the integrated isoform).

Isoform count was defined as the sum of IsoSeq read FL counts that were linked to a specific isoform but not to any other isoforms; we referred to this value as PBcount.

### Classification and filtering with SQANTI

We classified and filtered curated isoforms with SQANTI^27^. For classification, we used the genomic range of isoforms in GTF format, TPM of RNA-seq yielded by Salmon, and the number of FL reads summed in the last section. SQANTI uses random forest to determine whether an isoform is an artifact. As in the primary setting, isoforms with all splicing junctions matching those in annotated transcripts (full-splice match) were set as true positives in the training data; true negatives were defined as those transcripts with at least one novel and non-canonical splicing junction. We used isoforms that passed the SQANTI filter as our full-length transcript library for downstream analyses.

### Chimeric reads

Chimeric reads are mapped onto more than two genomic regions; however, because long reads yield a certain amount of sequencing error, there might be an uncertainty of several bp about the fusion sites. Therefore, when we found only one position with canonical splice junctions, as long as it was within the uncertainty range of the fusion sites, we selected the position as a fusion point. For reads with splice junctions as fusion points, we set an additional requirement that they have genomic breakpoints within 100,000 bp of the fusion points. For reads that were not confirmed, assuming that they had breakpoints on exonic regions, we set the requirement that there be genomic breakpoints within 100 bp of the fusion points. Of the three-piece fusion transcripts with two fusion points, we selected those with one linked to a genomic breakpoint, and the other one unlinked. Following that, we used blastn to manually search for possible genomic breakpoints that correspond with unlinked fusion points^66^.

### A simulator for long-read RNA sequencing

Although multiple long-read genomic sequence generators have been developed for simulation^67–69^, none were designed for cDNA sequencing. Therefore, we created a simulator for long-read RNA sequencing, simlady (SIMulator for Long-read transcriptome Analysis with RNA DecaY model). In contrast to long-read genomic sequencing, in which reads are generated from the distribution of read length, reads are generated from template transcripts in long-read RNA sequencing. The lengths of generated reads could be different from the original template transcripts; we have focused on RNA 5′ degradation and sequencing error as the main reason for this. RNA decay is a major reason why transcript start sites (TSS) of IsoSeq reads can be inaccurate^70^. On the other hand, for the positions of transcript termination sites, it has been reported that there are only a few amounts of error^24^. RNA decay was fitted by gamma distribution. The ‘pelgam’ function implemented in ‘lmom’ R package was used for fitting. A public dataset that uses MCF-7 cell lines for IsoSeq (https://github.com/PacificBiosciences/DevNet/wiki/IsoSeq-Human-MCF7-Transcriptome) is often used in simulator evaluation^68^; we also used this dataset for evaluation. Universal Human Reference (brain, liver, and heart) IsoSeq data (https://github.com/PacificBiosciences/DevNet/wiki/Sequel-II-System-Data-Release:-Universal-Human-Reference-(UHR)-Iso-Seq), another public dataset, was used for validation. We investigated how much the TSS of FLNC reads were shortened according to the nearest upstream TSS in GENCODE. Using the gamma distribution, the shortened length matched well below 10,000 bp (Supplementary Figs. 2a–d). This distribution was extremely heavy-tailed, and barely matched the fitted curve above 10,000 bp. One possible explanation for this is that because the GENCODE TSS annotation was imperfect, there were FLNC reads that incorrectly linked to distant reference TSSs. As of sequence error model, we used the SimLoRD^68^ model. SimLoRD inserts errors in order not to change the read length, but the read length changes actively according to the error inserted. Because SMRT sequencing data has a transcript length–dependent distribution, each read is sampled according to the probability derived from fold change and transcript length in order to reconstitute this distribution.

### Simulations with different settings

We generated two groups of short-read and long-read RNA-seq data from the GENCODE annotation, with the following parameters: number of samples per group, 8; fold change for DTU, 4; short-read length, 100 bp; short-read depth, 50,000,000 reads; and number of FLNC reads, 250,000. We changed these values one by one to investigate the effect on DTU inference (Supplementary Fig. 2). In detail, we defined the relative expression of all isoforms as 1 except for a randomly selected 10% of genes, for which we randomly selected 2 isoforms for DTU and assigned the pre-defined fold change value to one isoform in the first group and the other isoform in the second group. The ‘simulate_experiment_countmat’ function in the polyester R package^71^ was used for simulating short-read RNA-seq data; subsequently, the reads were shuffled because the reads were written out for each transcript. To simulate FLNC reads without specifying read length distribution, we used simlady, which generated reads under the log-normal distribution inherited from SimLoRD^68^. The FLNC reads were then processed to cluster reads by ‘isoseq3 cluster’ with the ‘–singletons’ option. Although IsoSeq3 discards singletons, we determined the number of FLNC read suitable for IsoSeq2, which additionally uses non-full-length reads and tolerates clusters with only one FLNC read. Therefore, we combined singletons with clustered reads and used them as input for MuSTA.

### Simulations based on the breast cancer dataset

We generated two groups of short-read and long-read RNA-seq data from the FSM and NIC isoforms in the MuSTA-derived transcriptome generated from 22 breast cancer specimens. The number of samples per group was 8 (short-read) and 14 (long-read), as in the original data. We permutated the log-averaged expression of FSM isoforms and NIC isoforms separately. We randomly assigned DGE for 25% of all genes, and DTU for 10% of all genes so that 4% of genes would be assigned as both DGE and DTU. These values were approximately the same as the original breast cancer data at an FDR threshold of 0.05. The expression fold change between groups was set to 4 for all isoforms of DGE genes, such that the log-averaged expression remained the same. As for DTU genes, we shuffled all genes and tried to select two DTU isoforms for which one isoform had higher expression in the first group and the other had higher expression in the second group. That is, we selected two isoforms with the highest expression randomly from the following so that the NIC rate against all DTU isoforms reached the pre-defined value: (i) two FSM isoforms, (ii) one FSM isoform and one NIC isoform, or (iii) two NIC isoforms. Again, we set the expression fold change between groups to 4 for DTU isoforms so that the log-averaged expression remained the same. Short-read and long-read RNA-seq reads were simulated as described above, with the exception that the length distributions of polished reads in breast cancer data were permuted and used for the FLNC read-length distribution.

### Differential gene expression

Differential gene expression was investigated with DESeq2^72^ as described in ref. ^73^. Isoform expression data obtained using Salmon were imported into R and summarized at the gene level using tximport^74^.

### Differential transcript usage

A comparison of state-of-the-art methods by Soneson et al.^11^ revealed that DEXSeq^75^ was most accurate. Therefore, we chose DEXSeq as the inference engine for differential transcript usage as described previously^73^. In brief, each isoform was treated as an exon, and a log-likelihood ratio test was performed under the setting with “~ sample + exon + subtype * exon” as the full model and “~ sample + exon” as the null model. To combine short-read RNA-seq data and full-length PBcount data, we concatenated both datasets and set “~ sample + exon + subtype * exon + data type * exon” as the full model and “~ sample + exon + data type * exon” as the null model. Note that this setting treated RNA-seq data and PBcount data derived from the same sample as biological replicates, whereas DEXSeq does not have a proper method for combining two technically replicated datasets with large batch effects, and we did observe a substantial difference between RNA-seq data and PBcount data. Although this could lead to an artificial increase in power, we obtained more conservative results from the concatenated data than from RNA-seq data in simulations (Fig. 1 and Supplementary Fig. 2). Gene-level and transcript-level false discovery rates were calculated with stageR^56^.

### Prefiltering of transcriptome

For pre-alignment prefiltering, we only retained those isoforms that had the first or second largest number of PBcount per gene in at least one sample and had PBcount ≥ 5 in all samples. Among these isoforms, up to 10 were chosen in descending order of PBcount. To avoid mis-mapping of RNA-seq, for each gene with no selected isoforms, we also retained one isoform with the largest PBcount. We defined the selected isoforms as “major” isoforms. For post-alignment prefiltering, we used the DRIMSeq^76^ filter and removed transcripts if their relative expression compared to the total expression of the related genes was lower than 0.1.

### Overlap between MuSTA-transcriptome and unannotated open reading frames

The list of high-confidence translated open reading frames identified in human induced pluripotent stem cells and human foreskin fibroblast was obtained from ref. ^29^. We lifted the positions from Hg19 to Hg38, and retained those that were uniquely lifted. We counted the number of open reading frames whose genomic ranges did not overlap with any GENCODE genes and were completely covered by MuSTA-transcriptome.

### Alternative splicing

We explored alternative splicing events of exon skipping/inclusion, alternative 5′, alternative 3′, mutually exclusive exons, and intron retention using the ‘generateEvents’ function of SUPPA2^77^. Next, we used the ‘performPCA’ function implemented in psichomics^78^ for principle component analysis of splicing events as described in the vignettes of the psichomics software (https://www.bioconductor.org/packages/release/bioc/vignettes/psichomics/inst/doc/CLI_tutorial.html).

### Domain prediction

We used HMMER^79^ to detect domains collected in Pfam^80^ (version 32.0) with the following command: - hmmscan –domtblout –noali -E 0.1 –domE 0.01 Pfam-A.hmm.

### Quantitative PCR and sequencing of fusion points

RNA was extracted from cells using the RNeasy Mini kit (Qiagen). Total cellular RNA was converted into cDNA by reverse transcription (SuperScript IV VILO Master Mix; Thermo Fisher) using random primers. Quantitative real-time PCR was performed using Power SYBR Green qPCR SuperMix-UDG with ROX (Thermo Fisher) on an Applied Biosystems PRISM 7900 Sequence Detection System. PCR conditions were as follows: 40 cycles of 95 °C for 15 s and 60 °C for 60 s. Complementary DNAs for fusion points were amplified by reverse-transcription PCR (RT-PCR) from RNA samples and subjected to Sanger sequencing. Primer sequences used in this study were as follows: IQCG-9-chr3-197892650-S: TGTGCTAAGTCACTGGCCTTTGTG; IQCG-9-chr3-197912810-AS: TGAGAACTTCTGATTCCCAGCCCT; IQCG-11-chr3-197892723-S: ATTTCTCCATCCAGAACTCCAGCC; IQCG-11-chr3-197914024-AS: GCTAACCTCAAGGACCAACTGCAA; TNS3-56-chr7-47304877-S: GAAGCAAAAGCCTGCTGAAAGGAG; TNS3-56-chr7-47328056-AS: AGCCCAAGGAGTTCCCTCTGTCT; TNS3-67-chr7-47304877-S: GAAGCAAAAGCCTGCTGAAAGGAG; TNS3-67-chr7-47344975-AS: GAGTCCATGTGTTCAACTCCAGCA; OGG1-Novel-Novel-S: AGAGGTGGCTCAGAAATTCCAAGG; OGG1-Novel-Novel-AS: CTTCCTTTCCCAGGCTCTTACCAA; SLC12A2_novel_SLC12A2-S: TTGGGGTATGGAGAGGAGCGTAAT; SLC12A2_novel_SLC12A2-AS: TGGCCACATTCCTATGATGAGC; HIST1H2AG_novel_ERVFRD-1-S: TGGAGTACAATGGTGTGATCTCGG; HIST1H2AG_novel_ERVFRD-1-AS: GTTCAGCCCTTGACTTGGGGTTTT.

### Chromatin modifications in breast normal/cancer cell lines

ChIP-seq of chromatin modifications for MCF-7, MDA-MB-468, and MCF-10A were carried out by Franco HL et al.^55^, and subsequently collected, mapped to hg19, and peak-called using ChIP-Atlas^81^. We used the mapped data and the peak data with a threshold of *q* < 10^−5^ and lifted them to hg38.

### Cell lines

Human embryonic kidney (HEK) 293T cells and the murine mammary carcinoma cell line EMT6 were obtained from the American Type Culture Collection (ATCC) and maintained in Dulbecco’s modified Eagle’s medium (DMEM)-F12 supplemented with 10% fetal bovine serum (FBS) (both from Life Technologies, Carlsbad, CA, USA). The human mammary gland epithelial cell line MCF10A was obtained from ATCC and maintained in DMEM-F12 supplemented with 5% (vol/vol) horse serum (Biowest, Nuaillé, France), recombinant human epidermal growth factor (20 ng/mL) (Peprotech, Cranbury, NJ, USA), bovine insulin (10 μg/mL) (Sigma-Aldrich, St. Louis, MO, USA), hydrocortisone (0.5 μg/mL) (Sigma-Aldrich), and cholera toxin (100 ng/mL) (Sigma-Aldrich). MC38 (mouse colon carcinoma) cell line was obtained from Kerafast (Boston, MA, USA) and maintained in DMEM (FUJIFILM Wako Pure Chemical Corporation, Osaka, Japan) supplemented with 10% FBS (Life Technologies). All cell lines were authenticated by the providers using karyotype, isoenzmes, and/or microsatellite profiling (short tandem repeat or simple sequence length polymorphism). Cultured cells were tested for mycoplasma contamination using the MycoAlert Mycoplasma Detection Kit (Lonza).

### Gene transduction

The coding sequences of genes were amplified by RT-PCR and inserted into the retroviral vector pMXs-ires-bsr. All cDNAs were verified by Sanger sequencing. To produce infectious viral particles, HEK293T cells were co-transfected with the indicated plasmids along with the packaging plasmids (Takara Bio, Kusatsu, Japan).

### In vivo mouse studies

Female C57BL/6J, BALB/c, and BALB/c-nu/nu mice (5–7 weeks of age) were purchased from Charles River Laboratories Japan (Yokohama, Japan) and used at 6–9 weeks of age. MC38 cells or EMT6 cells (1 × 10^6^ cells) infected with retroviral vector expressing *ERVFRD-1* or mock vector were subcutaneously inoculated into the flanks of the mice, and tumor size was monitored every 3 days. Tumor diameter was measured using calipers, and tumor volume was determined by calculating the volume of an ellipsoid using the following formula: length × width^2^ × 0.5. All mouse experiments were approved by the Animals Committee for Animal Experimentation of the National Cancer Center Japan. All experiments met the United States Public Health Service Policy on Humane Care and Use of Laboratory Animals.

### Statistics and reproducibility

Differential gene expression (DGE) was inferred using the Wald test under a negative binomial distribution implemented in DESeq2^72^. Differential transcript usage (DTU) was calculated by the log-likelihood ratio test implemented in DEXSeq^75^. Enrichment of gene ontologies was calculated by the hypergeometric test. Student’s *t*-test was used for testing the difference of tumor weight in mouse models. To test the association between percent-spliced-in of *TNS3* and prognosis, regression analyses under Cox proportional hazards models were performed for four indicators, overall survival (OS), disease-specific survival (DSS), disease-free interval (DFI), and progression-free interval (PFI)^82^, with race, sex, age at diagnosis, subtype, and *TNS3* expression (*Z*-score normalized) as covariates. For DGE and DTU in breast cancer data, a two-tailed *P* < 0.01 was considered statistically significant, based on our observations in the simulation studies. For DGE in *TNS3*-expressing MCF10A cells, genes with two-tailed *P* < 0.1 were used for the subsequent hypergeometric test. Otherwise, a two-tailed *P* < 0.05 was considered statistically significant. In situations involving multiple tests, the false discovery rate was calculated using the Benjamini and Hochberg method, except that stage-wise correction was applied for DTU with StageR^56^.

### Reporting summary

Further information on research design is available in the Nature Research Reporting Summary linked to this article.

## Supplementary information

Supplementary Information

Description of Additional Supplementary Files

Supplementary Data 1

Supplementary Data 2

Supplementary Data 3

Supplementary Data 4

Supplementary Data 5

Reporting Summary

## Data Availability

The raw sequencing data have been deposited in the Japanese Genotype-Phenotype Archive (https://www.ddbj.nig.ac.jp/jga/index-e.html) under accession number JGAS000095. The data set ID for the whole-genome sequence and RNA-seq is JGAD000095, and the dataset ID for Iso-seq is JGAD000457. Supplementary Data (the annotation of the MuSTA-derived transcriptome generated from 22 breast cancer specimens, the annotation of the novel predicted proteins, and the correlations of gene expression between intergenic genes and their neighbor genes) are available at Figshare^83^. TCGA data were obtained via cBioPortal (https://www.cbioportal.org/).

## Code availability

MuSTA and simlady are freely available at https://github.com/shinichinamba/MuSTA and https://github.com/shinichinamba/simlady, respectively, under the MIT License. The other bioinformatic tools used in this study are freely available and listed below: Genomon^60^ (version 2.6.0), STAR^61^ (version 2.5.2a), RSEM^63^ (version 1.3.1), Salmon^62^ (version 0.12.0), SMRTlink^18^ (version 5.1.0.26412), LoRDEC^64^ (version 0.9), Minimap2^65^ (version 2.12-r847-dirty), SQANTI^27^ (version 1.2), DRIMSeq^76^ (version 1.10.1), DESeq2^72^ (version 1.22.2), DEXSeq^75^ (version 1.28.3), tximport^74^ (version 1.10.1), stageR^56^ (version 1.4.0), HMMER^79^ (version 3.1b2), SUPPA2^77^ (version 2.3), psichomics^78^ (version 1.8.2), and BLAST^66^ (version 2.9.0+).
