# Supplementary material for: Transcript-targeted analysis reveals isoform alterations and double-hop fusions in breast cancer
Source: Commun Biol. 2021 Nov 22;4:1320. doi: 10.1038/s42003-021-02833-4 (PMC8608905; doi:10.1038/s42003-021-02833-4)
Supplement: Supplementary file 1 — Supplementary Information [file 42003_2021_2833_MOESM1_ESM.pdf]

Transcript-targeted Analysis Reveals Isoform Alterations and Double-hop Fusions in Breast Cancer

Shinichi Namba *et al.*

Supplementary Notes ***2***

Supplementary Note 1: Simulations in various conditions **2**

Supplementary Note 2: Comparison of MuSTA with other pipelines **4**

Supplementary Note 3: Repetitive elements in unannotated transcripts **5**

Supplementary Note 4: Functional analysis and potential localization of intergenic genes **7**

Supplementary Note 5: Co-expression between intergenic genes and their neighbor genes **8**

Supplementary Figures ***9***

Supplementary Figure 1 .... **9**

Supplementary Figure 2 .... **11**

Supplementary Figure 3 .... **13**

Supplementary Figure 4 .... **15**

Supplementary Figure 5 .... **16**

Supplementary Figure 6 .... **17**

Supplementary Figure 7 .... **18**

Supplementary Figure 8 .... **20**

Supplementary Figure 9 .... **22**

Supplementary Figure 10 .... **24**

Supplementary Figure 11 .... **25**

Supplementary Figure 12 .... **26**

Supplementary Figure 13 .... **28**

Supplementary Figure 14 .... **30**

Supplementary Figure 15 .... **32**

Supplementary Figure 16 .... **34**

Supplementary Figure 17 .... **36**

Supplementary References ***38***

Supplementary Notes

Supplementary Note 1: Simulations in various conditions

We evaluated the performance of MuSTA for DTU detection using simulation data (Supplemental Figs. 2a–d, Methods). For expression data, MuSTA acquires transcripts per million (TPM) from short-read RNA-seq and PBcount from SMRT sequencing. We investigated whether either or both of these two were adequate for DTU detection. Detection performance was best when using both in combination to detect DTU, with true positive rate (TPR) of 0.95 and false discovery rate (FDR) of 0.059 when the target FDR was set to 0.01 (Supplementary Fig. 2e). At the transcript level, detection performance was less satisfactory than at the gene level (TPR was 0.89 and FDR was 0.32).

Because IsoSeq can detect a large number of unannotated isoforms, we also calculated TPR and FDR for genes with more than 15 isoforms (Supplementary Fig. 2f). Although there was no significant change in sensitivity, specificity for genes with more than 15 isoforms was significantly lower than specificity for all genes. We excluded accidentally detected unannotated genes when calculating TPR and FDR for genes with more than 15 isoforms, and this was likely responsible for the increase in TPR.

Previous work showed that for detection of DTU, exclusion of isoforms with lower expression improves performance[1]. Therefore, we tried two kinds of prefiltering. First, we found that sensitivity was lower when we filtered out isoforms with low short-read coverage (post-alignment prefiltering) (Supplementary Fig. 2g). Second, a larger number of isoforms per gene could result in an increase in the number of misaligned short-read

fragments; therefore, we experimented with only isoforms with substantial PBcount values (major isoforms) in order to obtain short-read TPM (pre-alignment prefiltering). Although DTU detected in major isoforms had better specificity than DTU detected in full isoforms, the sensitivity was lower. In conclusion, according to our simulation, we obtained the best performance when not using prefiltering. We hypothesized that this result was due to the simulation method, in which we assigned DTU independently from isoform expression. Biologically valuable DTU should have relatively high expression among isoforms that belong to the same genes. Therefore, we examined the effect of prefiltering in simulations based on permutation of the breast cancer dataset, in which we assigned DTU to isoforms with the two highest expression levels (Methods). Nevertheless, we observed that prefiltering substantially decreased TPR regardless of NIC rates against all DTU isoforms (Supplementary Fig. 2q). Our results imply that although we did not examine the most suitable parameters for prefiltering, DTU inference was best performed without prefiltering when using a MuSTA-derived transcriptome.

Next, we examined the performance of DTU detection as a function of four conditions: (i) number of samples in each group, (ii) fold change in DTU isoforms, (iii) total short-read number, and (iv) total long-read number. Sensitivity was consistently high regardless of the number of samples in each group (Supplementary Fig. 2h). As the fold change in DTU increased, FDR for DTU detection in transcript level increased unexpectedly (Supplementary Fig. 2i). As the total short-read number increased, FDR for DTU detection at the transcript level again increased unexpectedly (Supplementary Fig. 2j). These contradictory declines in specificity were associated with an increase in the total short-read count and the difference between groups in the short-read count of isoforms not defined as DTU (Supplementary Figs. 2l–o). On the other hand, TPR showed little

change when long-read sequencing depth increased (Supplementary Fig. 2k). This may reflect that the increase in the number of isoforms detected in long-read RNA-seq was limited (Supplementary Fig. 2p). This number seemed to be saturated at around 180,000, reflecting the setting that all simulated transcripts were generated from 203,673 known transcripts in GENCODE. In summary, we concluded that DTU inference was best performed when we used both short-read expression and PBcount without isoform prefiltering.

Supplementary Note 2: Comparison of MuSTA with other pipelines

We conducted simulations to compare MuSTA with two pipelines, ToFU[2] and FLAIR[3]. FLAIR was developed exclusively for ONT Nanopore, but the authors claimed that FLAIR can be used for SMRT sequencing (https://github.com/BrooksLabUCSC/flair). In addition to the default settings of the two pipelines, we also used two modified settings for fair comparison: ToFU followed by SQANTI filtering, and FLAIR with the option "minimum supporting reads" of 1. We found that the detection power of simulated reads was almost the same between MuSTA and ToFU (Supplementary Fig. 3a), but different in that ToFU generated redundant isoforms, which were not observed for MuSTA (Supplementary Figs. 3b and c). There was even a case in which ToFU generated 22 isoforms corresponding to one GENCODE transcript. Subsequent SQANTI filtering was effective for filtering spurious reads but did not improve the redundancy of ToFU. When we included incomplete-splice match isoforms for analyses, we observed that a small number of isoforms was redundant in MuSTA (Supplementary Fig. 3d). However, this trivial redundancy was designed intentionally, as we prioritized the accuracy of distinction of the samples in which isoforms were detected. In MuSTA, we collapsed

redundant reads in two steps, intra-sample collapse and inter-sample collapse, and did not merge shorter reads to longer reads in inter-sample collapse unless all splice junctions were shared. This prevented alternative first exons from confounding the sample distinction. A representative example was *ESR1*, for which we found many unannotated isoforms, some of which were transcribed from minor upstream TSSs (Supplementary Fig. 4). When other strategies were used, major isoforms were considered to be 3' fragments of the isoforms transcribed from the minor upstream TSSs. Indeed, MuSTA was able to distinguish more accurately than ToFU whether samples expressed the simulated transcripts (Supplementary Fig. 3e). With FLAIR, the detection rate of the simulated transcripts was low (Supplementary Fig. 3a). Modification of the option "minimum supporting reads" only partially improved the rate. Hence, we excluded FLAIR from the comparison of sample distinction ability, as it collapses redundant isoforms without preserving sample-level information. These results were not affected by the number of simulated reads (Supplementary Figs. 5 and 6). We conclude that a pipeline designed for SMRT sequencing was needed, and that MuSTA is a suitable tool, as it has the advantages of low redundancy and accurate distinction among samples expressing isoforms.

Supplementary Note 3: Repetitive elements in unannotated transcripts

Although repetitive elements are difficult to investigate with short-read sequencing, they play important roles in mammalian genomes, such as transcription and chromatin regulation[4]. In particular, transposases have been repurposed to form new protein-coding genes[5]. Therefore, repetitive sequences might contribute to unannotated transcripts. We speculated that the long-read length of IsoSeq reads would enable us to deeply evaluate

this issue. Using RepeatMasker[6], we scanned repetitive sequences for six categories of unannotated transcripts, excluding NIC. We found 871 genes that were intergenic based on the GENCODE transcriptome but overlapped with other genes in the MuSTA transcriptome, indicating that some gene regions were expanded in the MuSTA-transcriptome. Therefore, we removed these genes in subsequent analyses, yielding a total of 2,619 intergenic genes. NNIC transcripts contained repetitive elements in 8.5% of their sequence length, but repetitive elements were detected in 62.0% sequences of intergenic transcripts (Supplementary Fig. 8). Among repetitive elements, LINEs, LTRs, and SINEs occupied the largest proportions of intergenic transcripts. Although satellite sequences were rarely observed, they ranged over 60% of transcript length, as in the intergenic transcripts in which satellite sequences were detected. Given the high occupancy of repetitive elements in the intergenic transcripts, we suspected that reads containing repetitive sequences were mistakenly mapped to intergenic regions. We repeated the same analyses using the original IsoSeq reads that were uniquely linked to intergenic genes, and obtained similar results (Supplementary Fig. 9). Additionally, we examined the secondary alignments of reads generated by minimap2[7], but only 5 out of 2,906 reads had secondary alignments. Furthermore, we used another mapping tool, Magic-BLAST[8], to verify the accuracy of minimap2 mapping. Although the mapping performance of minimap2 is superior to that of Magic-BLAST for SMRT-sequencing transcript reads, Magic-BLAST uses a BLAST-like local alignment algorithm and removes frequently detected seed sequences before alignment. Minimap2 can also skip seed sequences containing repetitive sequences. All except for eight reads were mapped to the same loci by Magic-BLAST and minimap2. These results indicated that we took advantage of long

read length to successfully map reads containing repetitive sequences using long-read aligners.

Supplementary Note 4: Functional analysis and potential localization of intergenic genes

We used a homology-based model, blastx[9], and queried the intergenic transcripts against a protein database (NCBI non-redundant protein sequences restricted to human proteins, from which we removed protein models [XM/XP]). We combined the results with the protein domains that matched the intergenic transcripts detected by InterProScan[10]. Subsequent Gene Ontology analyses with Blast2GO[11] showed that intergenic transcripts were associated with molecular interactions and involved in metabolic processes (Supplementary Figs. 10 a–c). We also predicted potential localization of 154 intergenic genes that were predicted to code proteins longer than 50 aa. Hum-mPLoc 3.0[12] predicted that 86 proteins localized in the nucleus and 52 localized in the cytoplasm (Supplementary Fig. 10d). We also estimated the potential localization of the intergenic transcripts at the mRNA level using DM3Loc[13]. DM3Loc is a deep learning model that predicts localization to six compartments: exosome, ribosome, nucleus, membrane, endoplasmic reticulum, and cytosol. Localization was predicted for 54.8% (1,574/2,873) of the transcripts, although 86.0% (14,591/16,967) of FSM transcripts were predicted to localize in at least one compartment. The lower prediction rate might be explained by the length of the intergenic transcripts (Supplementary Fig. 10e). In addition, the ratio of repetitive elements was slightly higher in the unpredicted transcripts (Supplementary Fig. 10f). For both FSM and intergenic transcripts, the largest number of transcripts localized in the exosome;

surprisingly, however, intergenic transcripts were more likely to localize in the cytosol than in the nucleus or other compartments (Supplementary Fig. 10g). Membrane was the compartment with the smallest number of the localized intergenic transcripts. Together, these findings indicate that intergenic transcripts might be involved in different cellular functions than the FSM transcripts.

Supplementary Note 5: Co-expression between intergenic genes and their neighbor genes

As we discussed in relation to gene fusion events (Fig. 6), intergenic genes could influence the expression of their neighboring genes through *cis*-regulatory elements. Therefore, we sought to determine whether expression of intergenic genes and their neighboring genes was correlated. We constructed three types of neighboring protein-coding gene sets: head-to-head antisense genes, upstream genes in the same strand, and downstream genes in the same strand. We evaluated Pearson's correlation of gene expression and observed a heavy tail of positive correlation for all three gene sets (Supplementary Fig. 11a). Because many intergenic transcripts contained repetitive sequences (Supplementary Figs. 8 and 9), the expression of intergenic transcripts might be biased. Therefore, we also calculated Pearson's correlation using PBcount, the number of uniquely associated IsoSeq reads, and confirmed that the heavy tail was unchanged (Supplementary Fig. 11b). To measure the impact of repetitive sequences on co-expression, we restricted the analyses to intergenic genes for which repetitive sequences were detected in more than 90% of the sequence (Supplementary Figs. 11c and d). The heavy tail of positive correlation was still observed, and we concluded that there were co-expression patterns for intergenic genes and their neighboring genes, regardless of the

presence or absence of repetitive sequences. These results indicated that genes originating from repetitive sequences were involved in regulation of expression by local genome architecture. Notably in this regard, we observed remarkably high positive correlations for head-to-head genes with distances of less than 1,000 bp. This suggests that some intergenic genes were promoter-associated noncoding RNAs transcribed from bidirectional promoters. In summary, we estimated the potential functions and localization of the intergenic genes and revealed the regulation of the expression of genes containing repetitive elements. Together, the results will contribute to further characterization of unexplored genes.

Supplementary Figures

Supplementary Figure 1

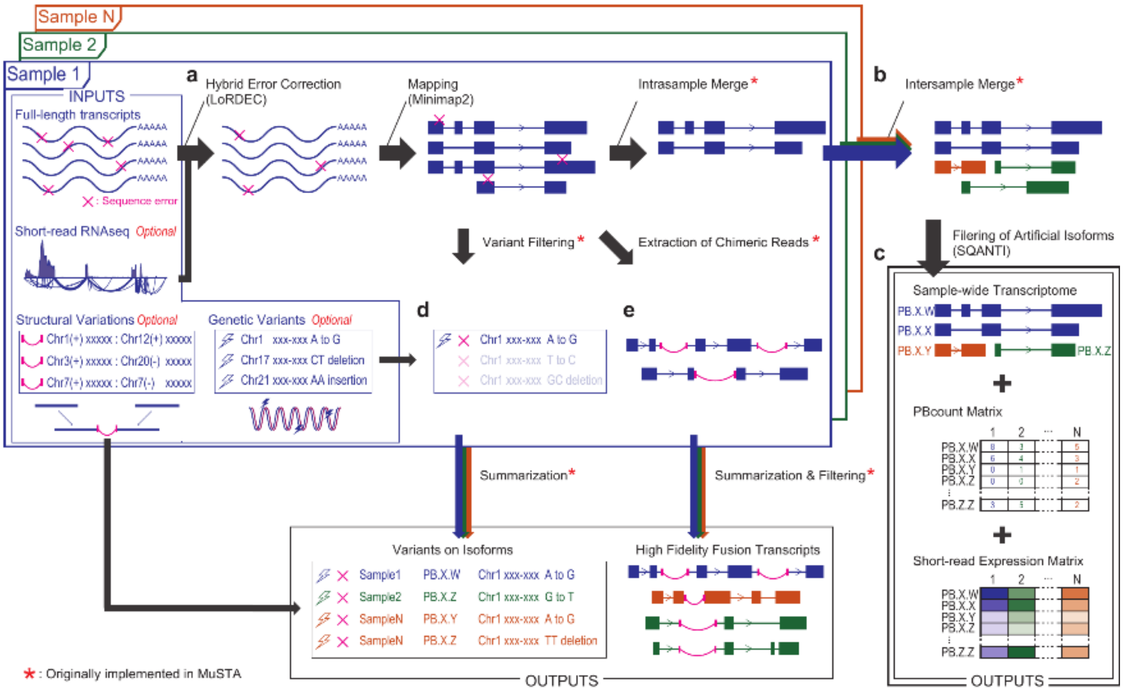

A schematic view of the MuSTA workflow. a–c, IsoSeq cluster reads go through hybrid error correction if the user selects this option; otherwise, this step will be skipped (**a**). Next, those reads are mapped to a reference genome using minimap2. Reads mapped to a single genomic region are merged into non-redundant isoforms in two consecutive steps, intra-sample (**a**) and inter-sample (**b**). Short-read RNA-seq data is aligned to the isoforms, and a transcripts per million (TPM) value is calculated for use in the SQANTI filter (**c**). SQANTI classifies the merged isoforms by comparison with a reference transcript annotation and excludes artificial isoforms with a random forest algorithm. In each sample, and for each original cluster read, the number of full-length non-chimeric (FLNC) reads is summed. This process is limited to cases in which the original cluster read is linked to only one isoform. The number of FLNC reads was named "PBcount." Then, short-read TPM is again calculated for the SQANTI-passed isoforms. **d**, The original reads contain sequence mismatches against the genome, and these mismatches are also linked to the merged isoforms. The mismatches can be filtered with user-specified genomic variants. Finally, the SQANTI-passed isoforms are reported with information about PBcount, short-read TPM, and sequence mismatches of original reads. **e**, For reads that are separately mapped to multiple genomic regions, their genomic positions and mismatches against the reference genome are summarized. Of these chimeric reads, transcripts consisted of splicing junctions from the reference transcript annotation or SQANTI-passed isoforms are selected and reported. Furthermore, given the structural variation data, MuSTA categorizes transcripts according to the presence or absence of associated structural variations. All of the procedures using short-read RNA-seq data or variation data are optional, and users can execute MuSTA as long as they have IsoSeq reads.

Supplementary Figure 2

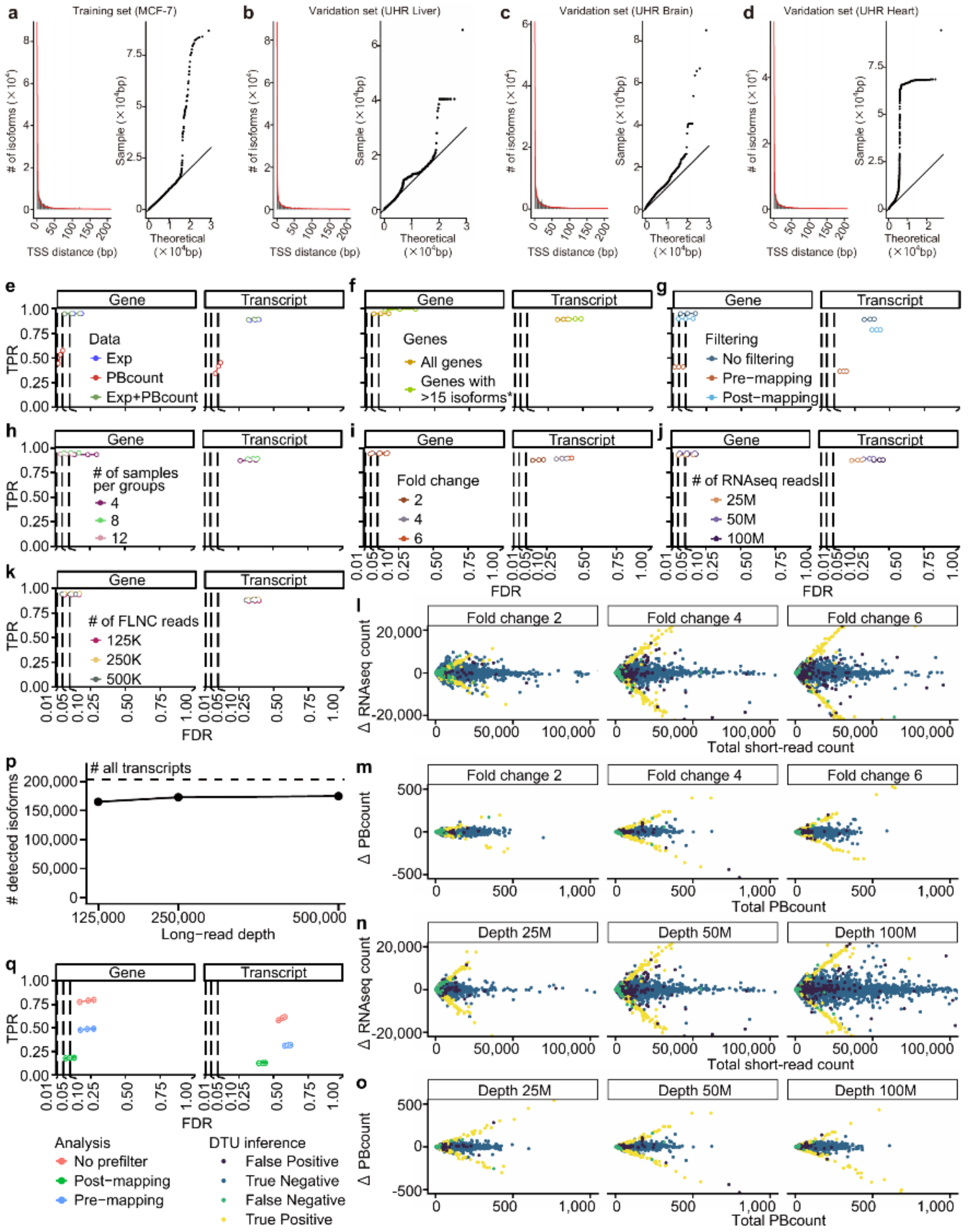

Simulation results under different conditions. a–d, Parameter estimation for RNA 5’ degradation. Figure **a** shows the training data set, and figures **b–d** show validation data

sets. Left: Distance between the TSS of FLNC reads and the nearest upstream TSS in GENCODE TSS. The red line shows the fitted curve. Right: Quantile–quantile plot of curve fitting. TSS, transcript start site; FLNC, full-length non-chimeric. **e–k**, True positive rate (TPR)–false discovery rate (FDR) plots of differential transcript usage (DTU) simulations. The dots on each plot indicate the target FDRs of 0.01, 0.05, and 0.1. Conditions were changed according to expression data (**e**), number of isoforms (**f**), isoform prefilter (**g**), number of samples in each group (**h**), fold change in DTU isoforms (**i**), and depth of short-read (**j**) and long-read (**k**) RNA sequencing. Note that we excluded accidentally detected unannotated genes when calculating TPR and FDR for genes with more than 15 isoforms (asterisk). **l–o**, The sum and difference in expression between groups when (**l** and **m**) changing the fold change and (**n** and **o**) changing the depth of short reads. In **l** and **n**, expression data were short-read counts, whereas in **m** and **o**, they were PBcount values. **p**, The number of isoforms detected as a function of the depth of long reads. **q**, TPR-FDR plots of DTU simulations based on the permutation of the breast cancer dataset. NIC rates against all DTU isoforms were set to 0, 0.25, 0.5, 0.75, or 1; the results obtained using NIC rates of 0.25 are shown. The dots represent the mean, and the error bars the standard error, of three independent simulations.

Supplementary Figure 3

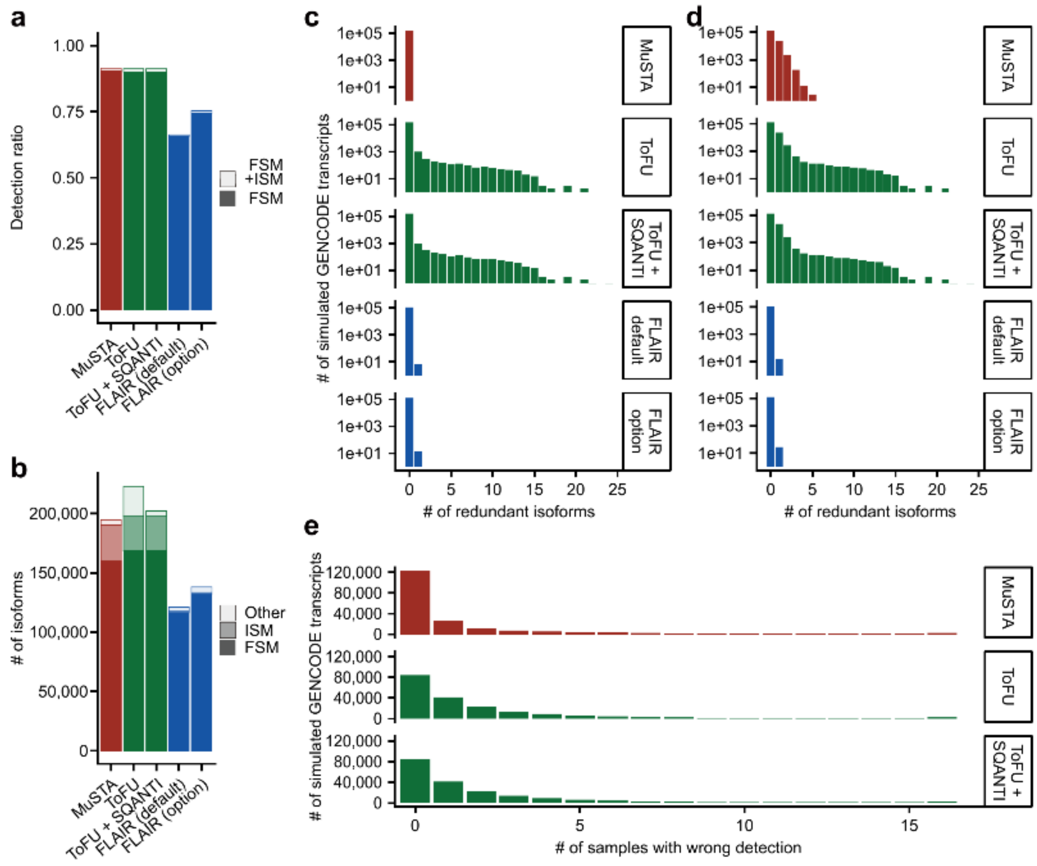

Performance comparison with other pipelines. We show representative results obtained using 250,000 simulated long reads, as described in the "Simulations with different settings" section of the Methods. Other results can be found in Supplementary Figs. 5 and 6. **a**, Rate of transcripts detected by each method relative to the GENCODE transcripts used in the simulation. Colored bars represent the rate restricted to FSM isoforms, and uncolored bars representing the rate when ISM isoforms were included. FSM, full-splice match; ISM, incomplete-splice match. **b**, Number of isoforms in the transcriptome constructed by each method. **c** and **d**, Number of redundant isoforms corresponding to GENCODE transcripts that have been already linked to another isoform.

The analyses were conducted for FSM and ISM isoforms (**c**) or restricted to FSM isoforms (**d**). The *y* axis is shown in a logarithmic scale. **e**, Number of samples in which detection of GENCODE transcripts did not match their existence (i.e., the GENCODE transcripts were used for the simulation but not detected, or vice versa). Both FSM and ISM isoforms were used for the analyses.

Supplementary Figure 4

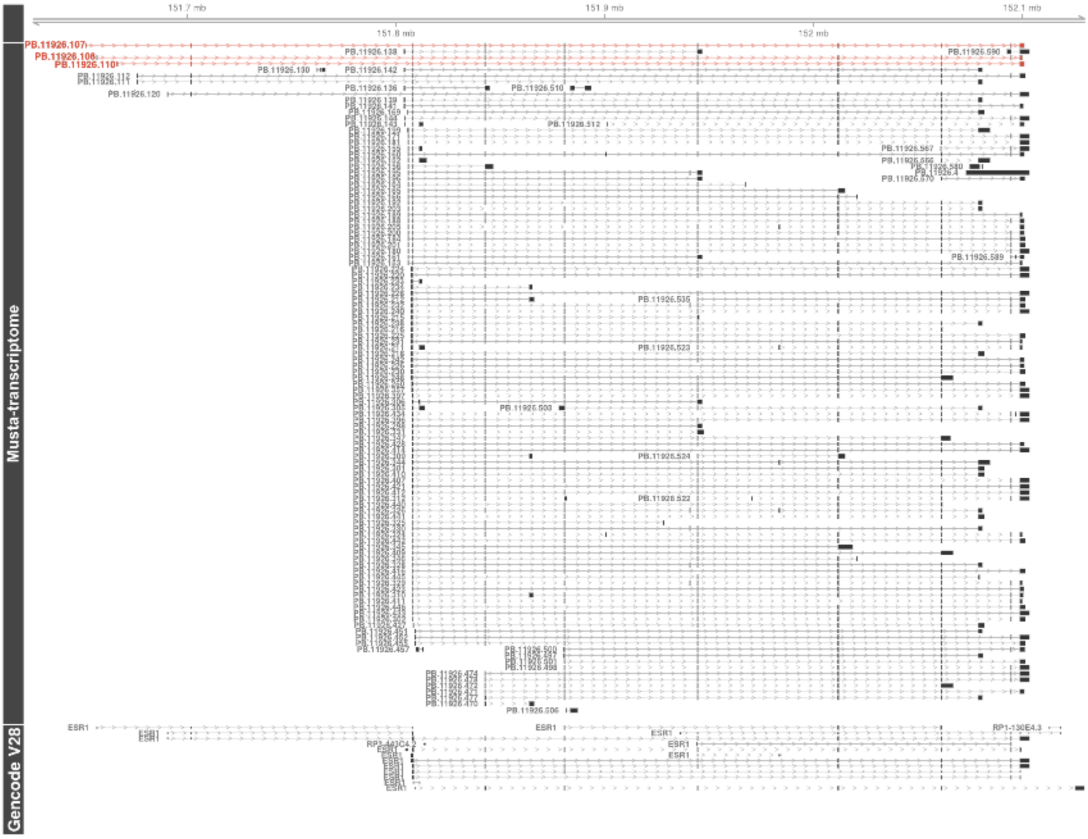

Multi-exonic isoforms in the *ESR1* region. From top to bottom, areas represent genome axis, MuSTA isoforms, and GENCODE annotation. Mono-exonic isoforms were omitted for visualization purposes. Three MuSTA isoforms transcribed from the most upstream transcription start sites are highlighted in red.

Supplementary Figure 5

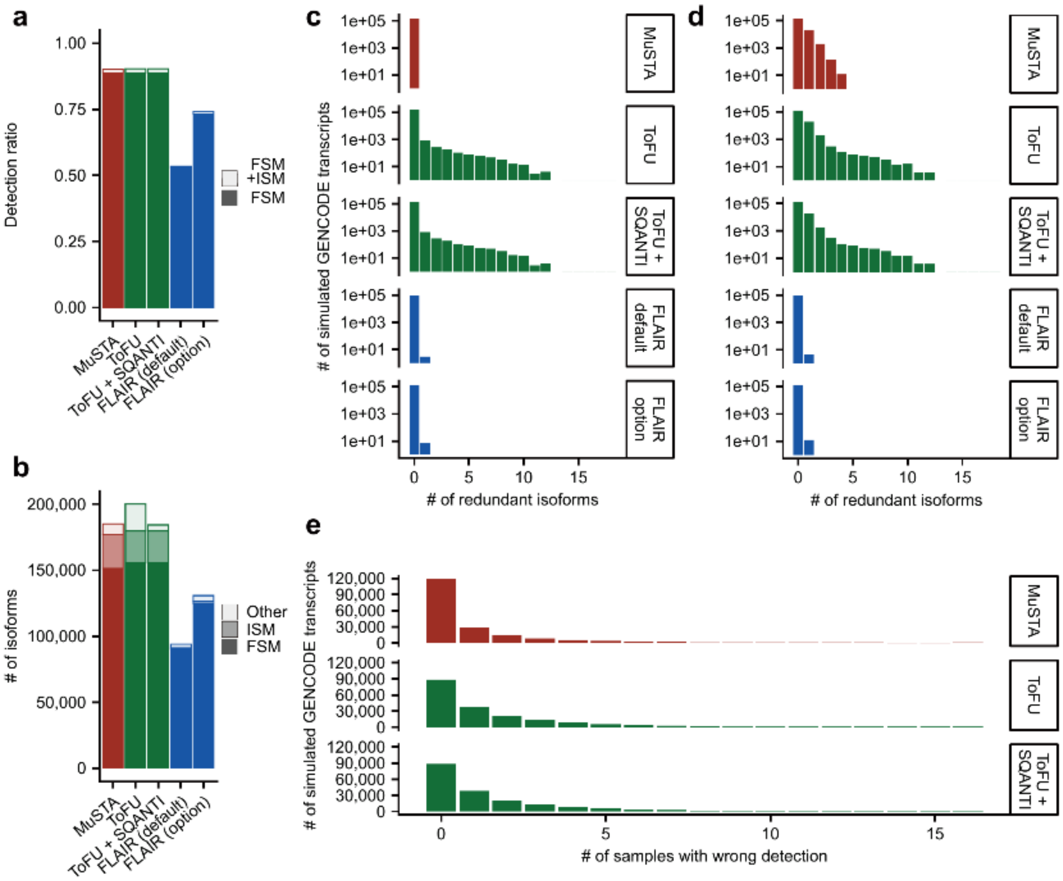

Comparison of performance with other pipelines, using 125,000 simulated long reads. Data display is described in the legend for Supplementary Fig. 3.

Supplementary Figure 6

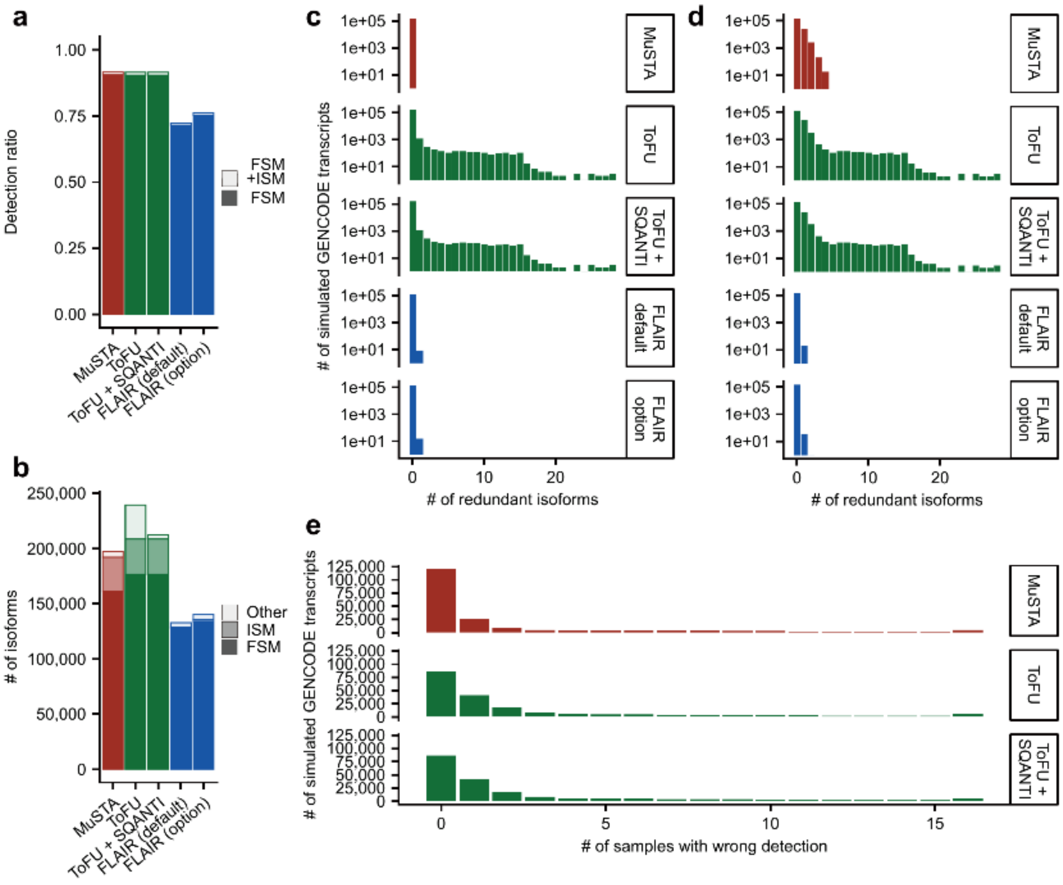

Comparison of performance with other pipelines, using 500,000 simulated long reads. Data display is described in the legend for Supplementary Fig. 3.

Supplementary Figure 7

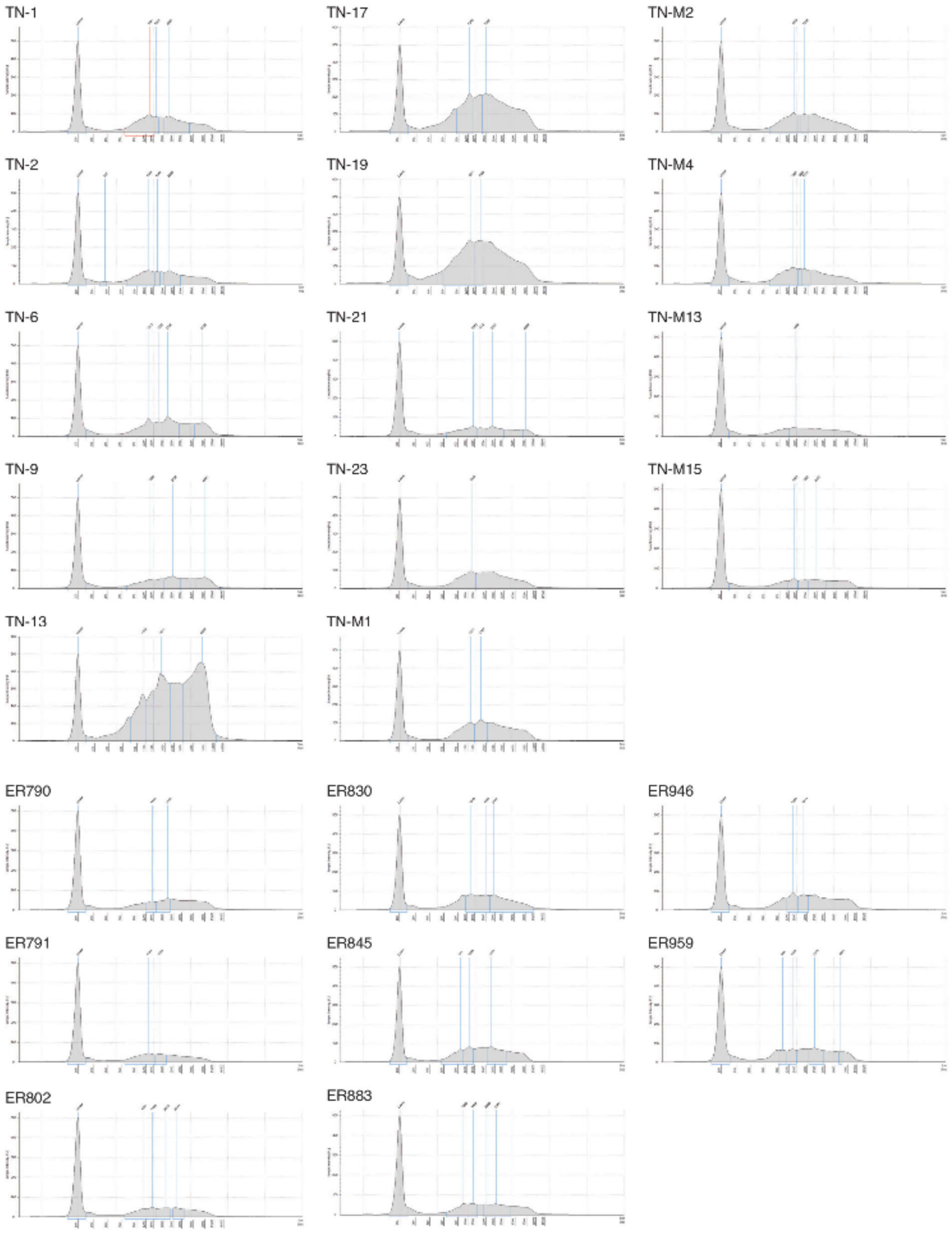

Distribution of read length resulting from SMRT sequencing of 22 clinical breast cancer specimens.

Supplementary Figure 8

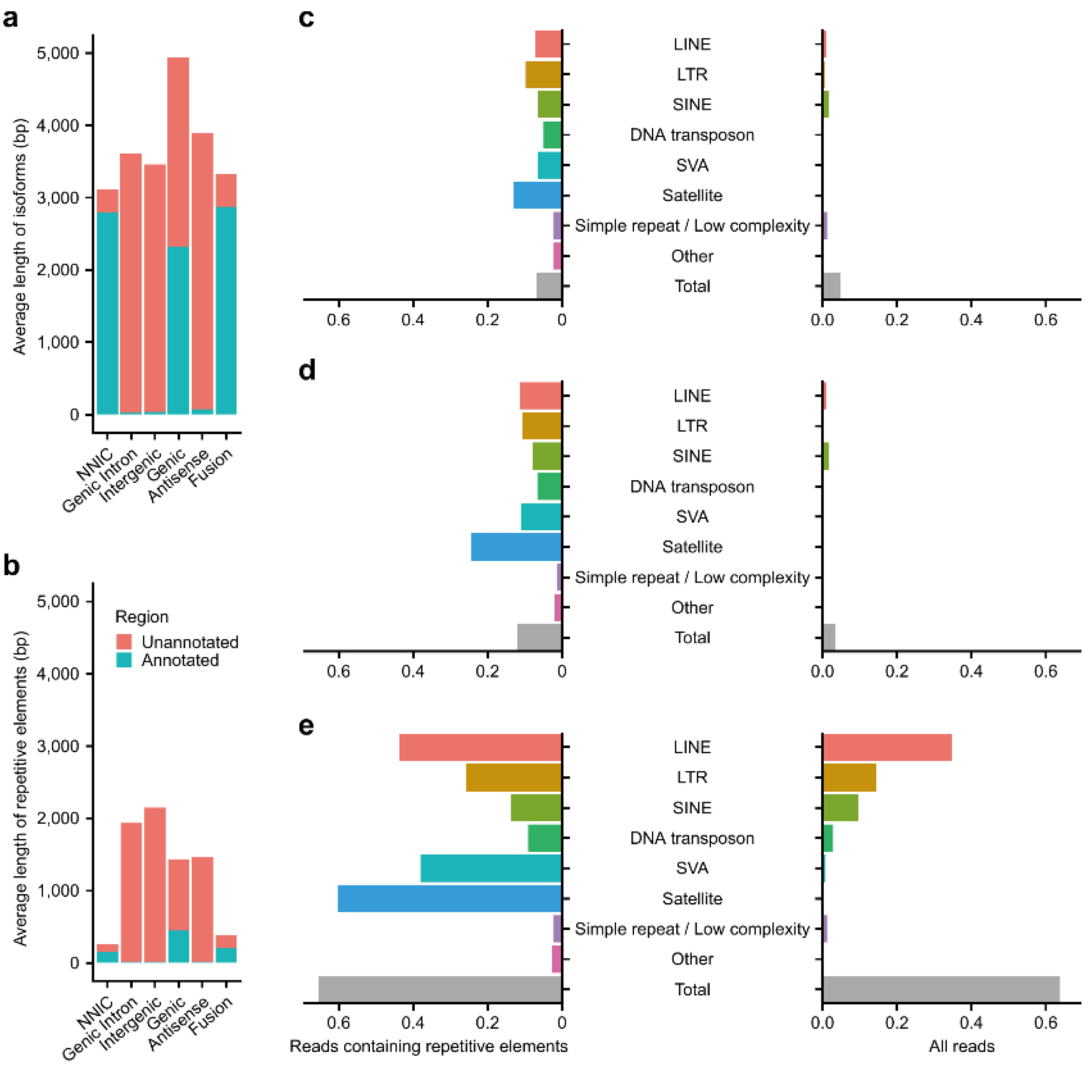

Repetitive elements detected in unannotated isoforms. a, Average length of isoforms for each SQANTI category, colored according to the fraction of isoform length overlapping the exonic regions of annotated isoforms in the GENCODE or MuSTA transcriptome. **b**, Average length of isoform segments in which repetitive elements were detected. **c**–**e**, Fraction of segment length with repetitive elements vs. total isoform length for novel not in catalog (NNIC) isoforms restricted to annotated regions (**c**), NNIC isoforms restricted to unannotated regions (**d**), and intergenic isoforms (**e**).

Bars represent the fraction of all isoforms (left) and the fraction of the subset of isoforms in which repetitive elements of the relevant class were detected (right).

Supplementary Figure 9

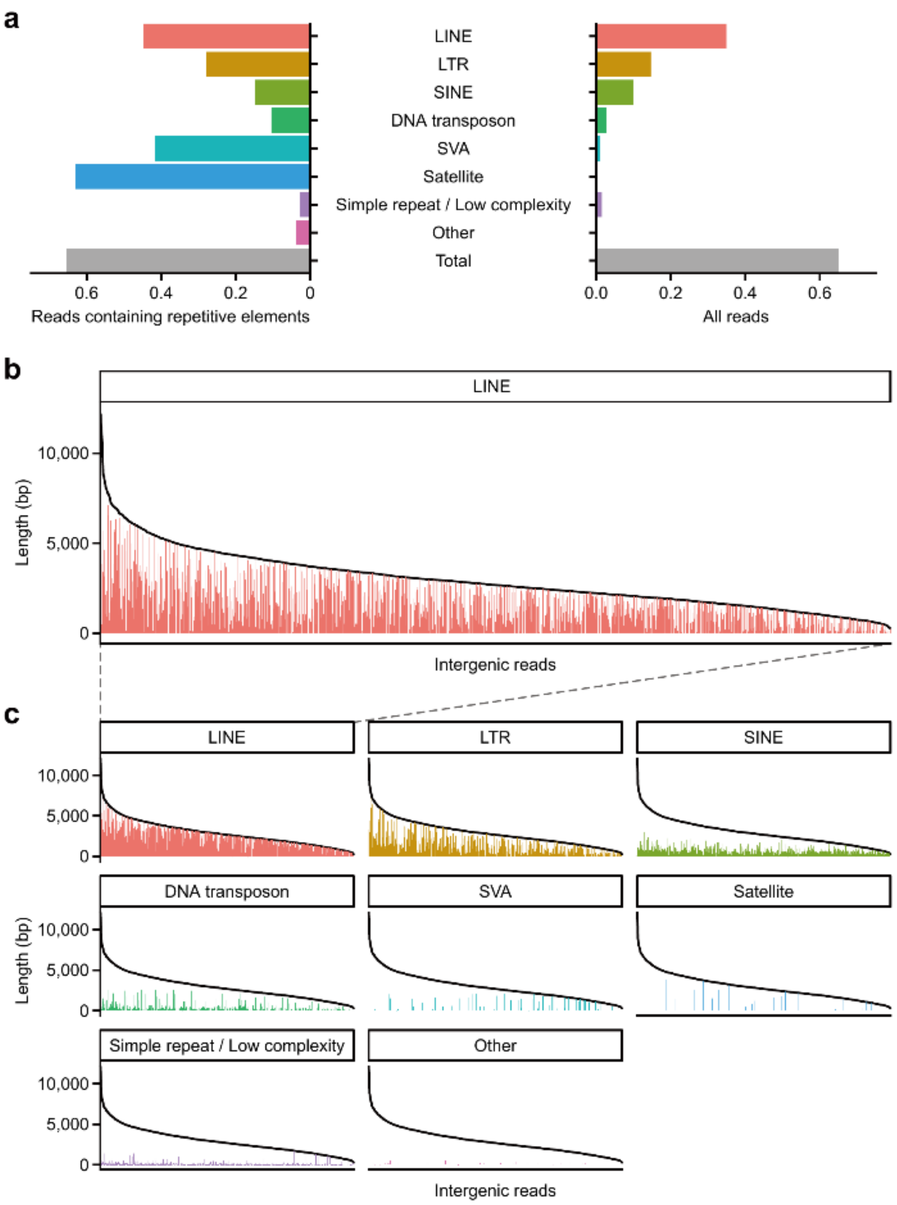

Repetitive elements detected in original reads of intergenic isoforms. a, The same plot as in Supplementary Fig. 8e for original reads of intergenic isoforms. **b** and **c**, Segment length with repetitive elements in each original read for LINEs (**b**) and eight classes of repetitive elements including LINEs (**c**). LINE, long interspersed nuclear

element; SINE, short interspersed nuclear element; LTR, long terminal repeat; SVA, SINE-VNTR-Alu.

Supplementary Figure 10

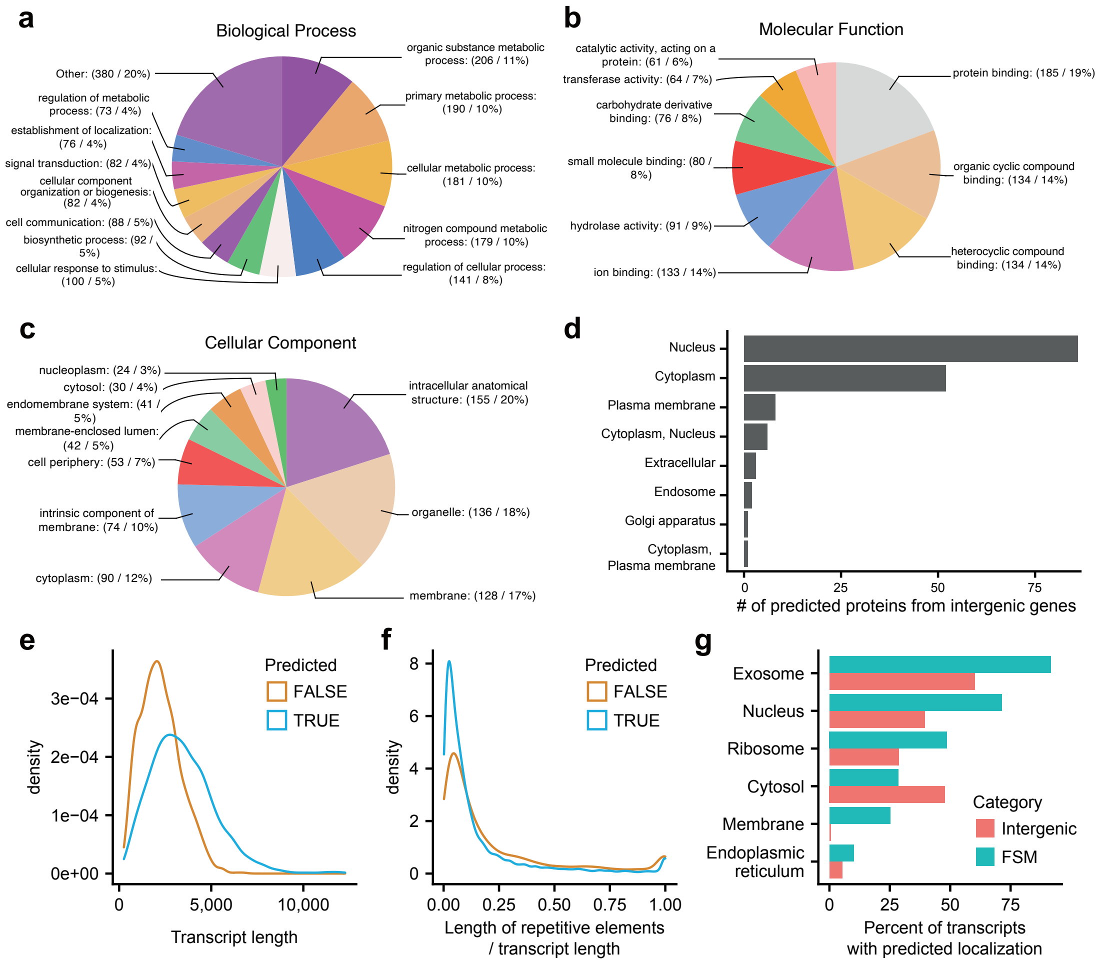

Functional analyses and predicted localization of intergenic genes. a–c, Gene ontology for biological process (**a**), molecular function (**b**), and cellular component (**c**). **d**, predicted protein localization for the intergenic genes that were predicted to encode proteins longer than 50 aa. **e** and **f**, density plots of transcript length (**e**) and ratio of repetitive elements (**f**) for intergenic transcripts. Colors represent whether mRNA level localization was predicted for at least one component. **g**, Predicted mRNA localization of intergenic transcripts and full-splice match (FSM) transcripts. Transcripts could be predicted to localize in more than one component.

Supplementary Figure 11

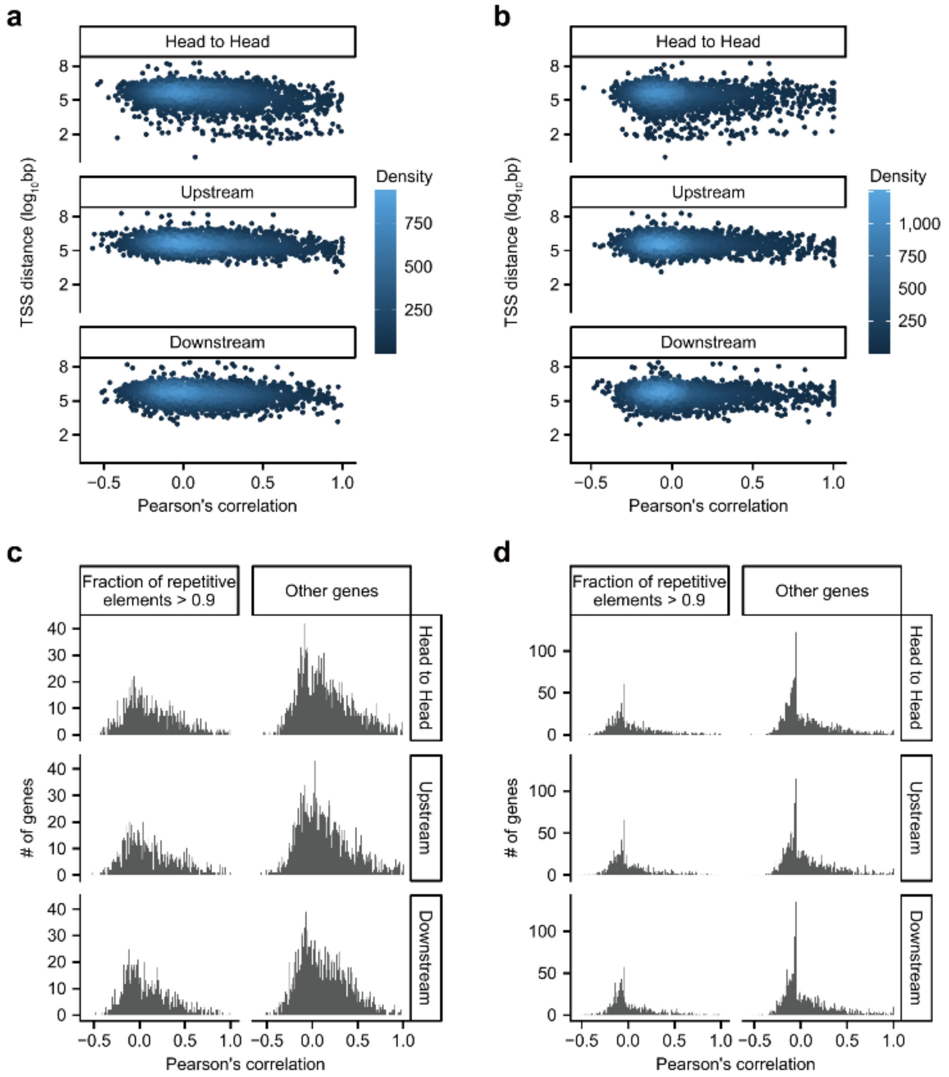

Co-expression patterns between intergenic genes and their neighboring genes. a and **b**, Pearson's correlation of expression and distance of transcription start sites (TSSs) between intergenic genes and their neighbor genes. TPM and PBcount were used for Pearson's correlation in **a** and **b**, respectively. **c** and **d**, Pearson's correlation of TPM (**c**) and PBcount (**d**) as in **a** and **b**, but stratified by the fraction of repetitive elements in the intergenic genes.

Supplementary Figure 12

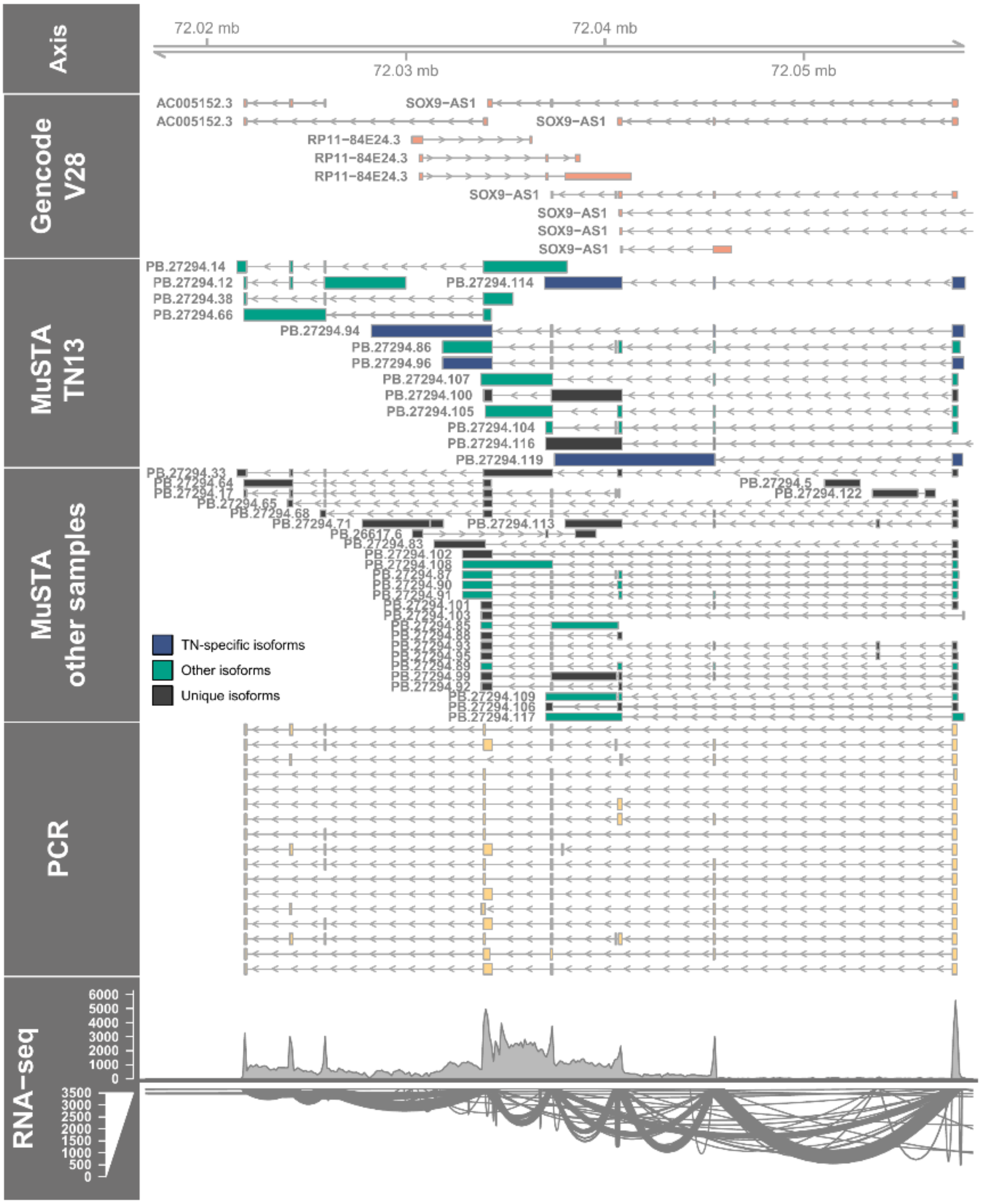

Nested PCR validation of *SOX9–AS1/AC005152.3* readthrough transcripts. From top to bottom, the areas represent genome axis, GENCODE annotation around *SOX9–AS1* and *AC005152.3*, MuSTA isoforms detected in sample TN13, MuSTA isoforms detected

in samples other than TN13, nested PCR products in TN13, and coverage and sashimi-plot of RNA-seq in TN13.

Supplementary Figure 13

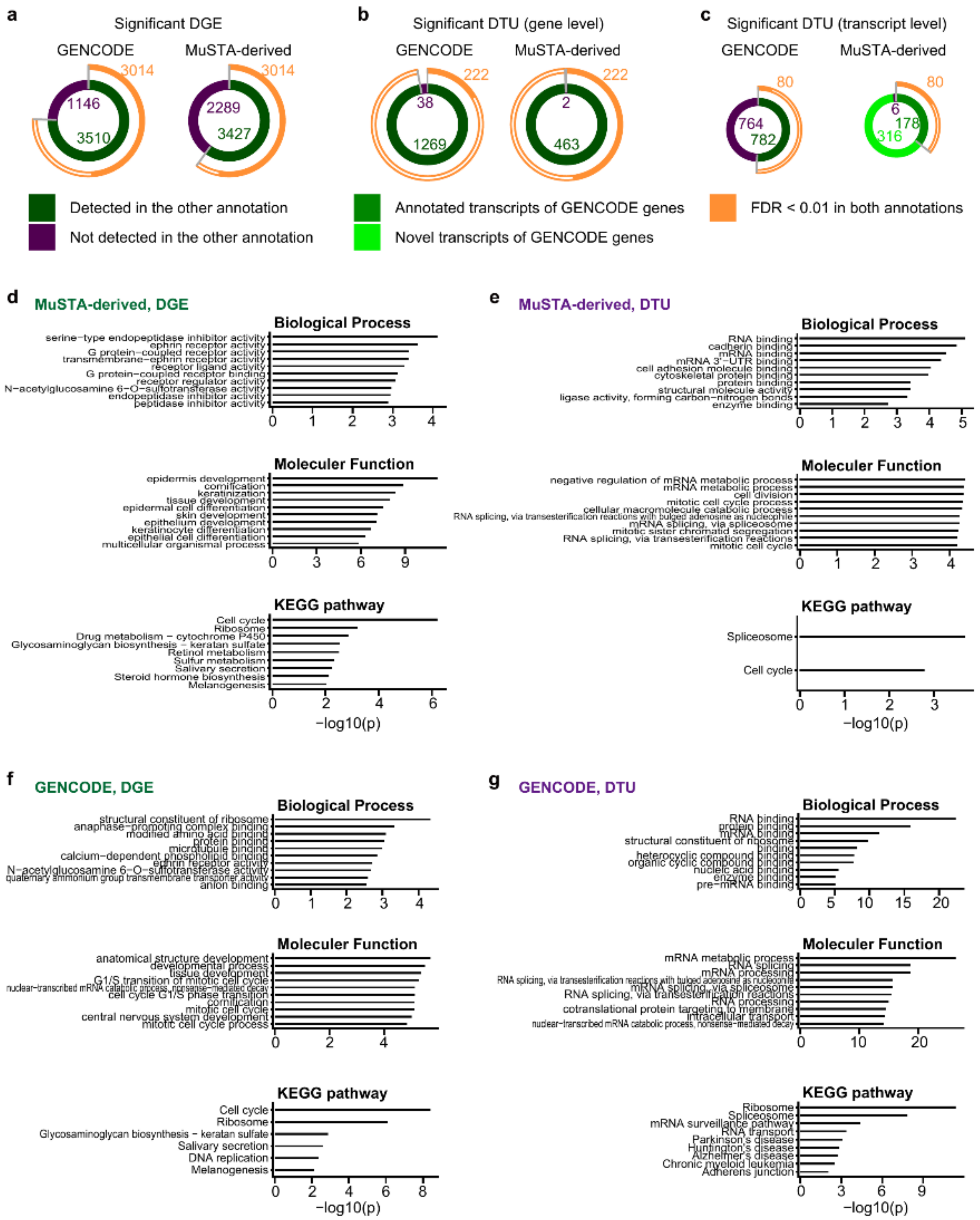

Composition of DGE and DTU genes, and Gene Ontology and KEGG pathway enrichment analysis in DGE and DTU genes. a–c, Donut plots representing genes with

DGE (**a**), genes with DTU (**b**), and isoforms with DTU (**c**). Left, DGE genes, DTU genes, and DTU isoforms detected with the GENCODE transcriptome. Right: DGE genes, DTU genes, and DTU isoforms detected with the MuSTA-derived transcriptome. Outer rings show the proportion of genes or isoforms that were labeled as DGE/DTU in both annotations with FDR < 0.01. Numbers represent the number of genes or isoforms in each group. **d** and **e**, Gene enrichment analysis for DGE (**d**) and DTU (**e**) genes for the MuSTA-derived transcriptome. **f** and **g**, Gene enrichment analysis for DGE (**f**) and DTU (**g**) genes for the GENCODE transcriptome. P-values were calculated by hypergeometric test. Top: Gene Ontology Biological Process (GO BP); center, Gene Ontology Molecular Function (GO MF); bottom, KEGG pathway.

Supplementary Figure 14

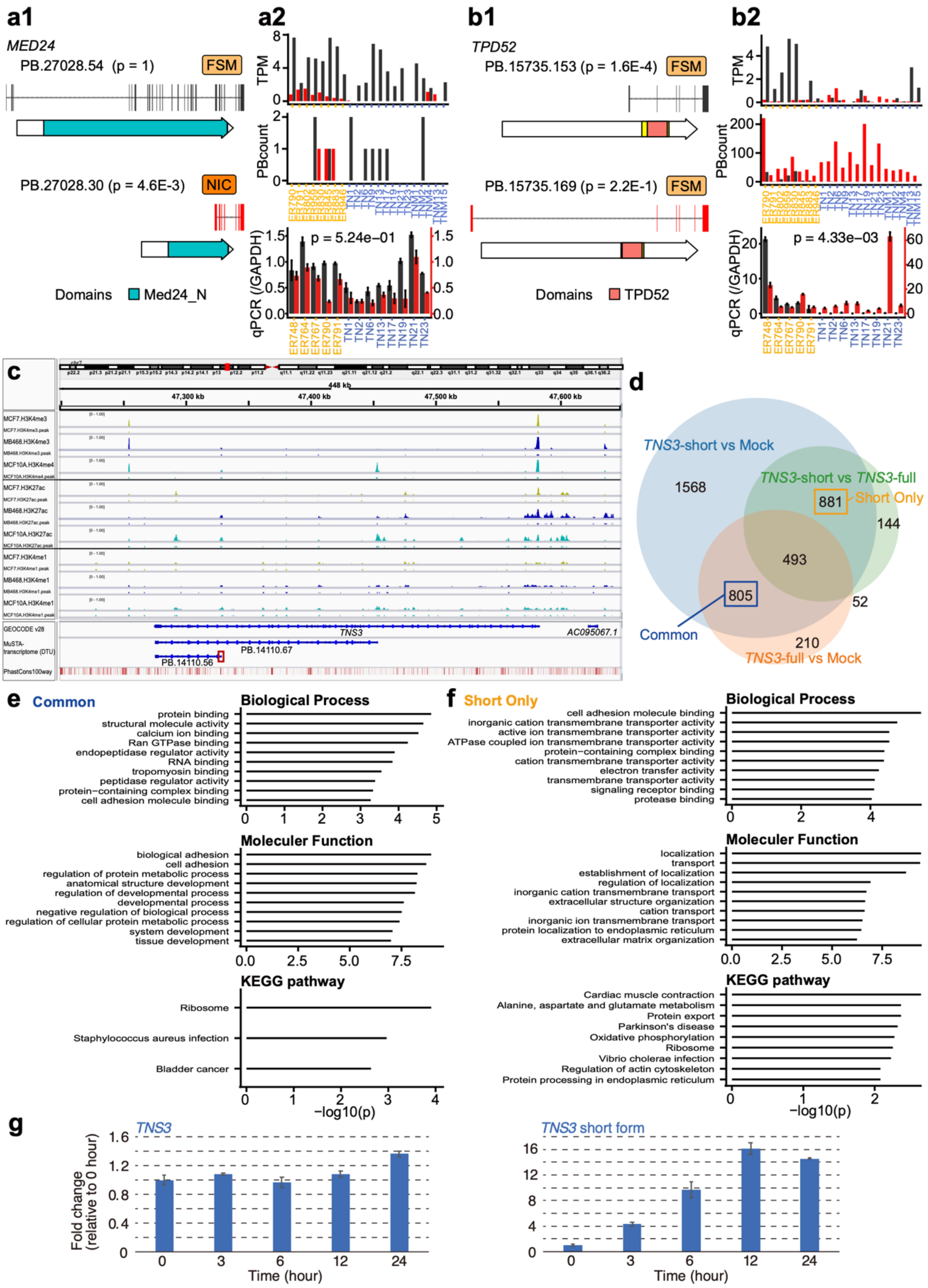

Differential transcript usage. Isoform description and expression for *MED24* (**a**) and *TPD52* (**b**). Shown are SQANTI classifications, transcript structures, predicted protein domains of two DTU isoforms with the smallest p-values. and expression of DTU isoforms. Three types of expression data are shown (i.e., transcripts per million (TPM) aligned to the MuSTA-derived transcriptome, PBcount, and qPCR expression normalized against *GAPDH*). In Figs. 4b and c, relative qPCR expression has two y-axes along with DTU isoforms, as qPCR was conducted separately for each isoform. Error bars in qPCR expression indicate the standard error of three replication studies. P-values for relative expression of DTU isoforms were calculated with two-tailed Mann–Whitney U test. **c**, Snapshots of Integrative Genomics Viewer showing chromatin modifications of three breast cell lines and evolutionary conservation in *TNS3* region. Shown are three chromatin modifications [H3K4me3 (enriched at promoters), H3K27ac1, and H3K4me1 (enriched at enhancers)] in MCF-7 (ER-positive breast cancer), MDA-MB-468 (TNBC), and MCF-10A (normal breast epithelium). **d**, Venn diagram representing three types of differential gene expression analyses using RNA-seq data of MCF10A cells with TNS3* isoform expression. Numbers denote the number of significantly affected genes (Benjamini–Hochberg-adjusted FDR < 0.1). **e** and **f**, Gene Ontology and KEGG pathway enrichment analysis for genes affected by both full-length *TNS3* and the *TNS3* short form (**e**) and genes affected exclusively by the *TNS3* short form (**f**). **g**, Expression of *TNS3* isoforms in the presence of 10 ng/mL TGFβ1. Mean qPCR measurements of three biological replicates are shown, normalized against *GAPDH*. Error bars represent standard error.

Supplementary Figure 15

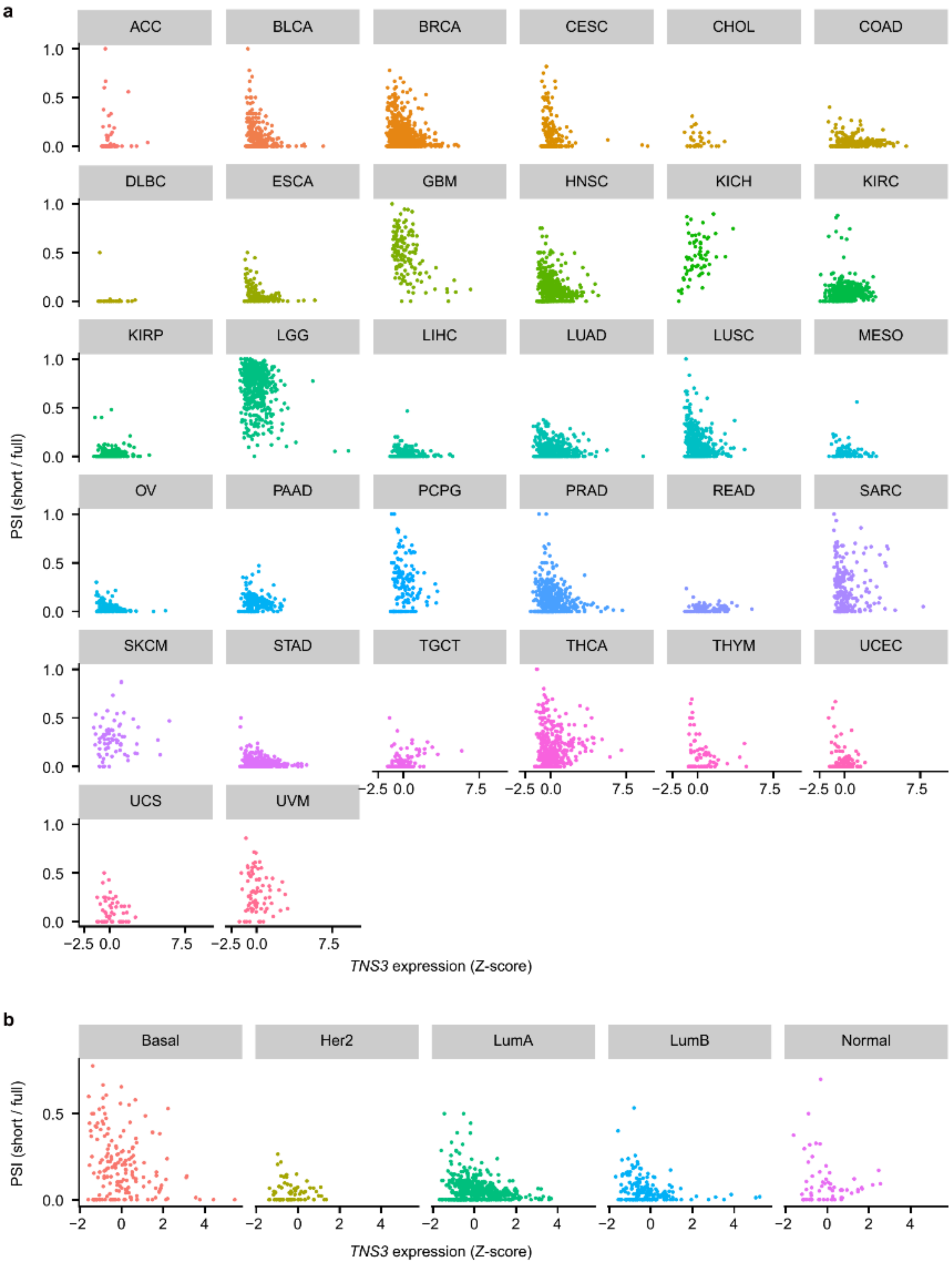

Expression of the *TNS3* short form in the Cancer Genome Atlas. Percent-spliced-in (PSI) of the first intron of the *TNS3* short form relative to the corresponding intron of full-length *TNS3* per cancer type (**a**) and per breast cancer subtype (**b**).

Supplementary Figure 16

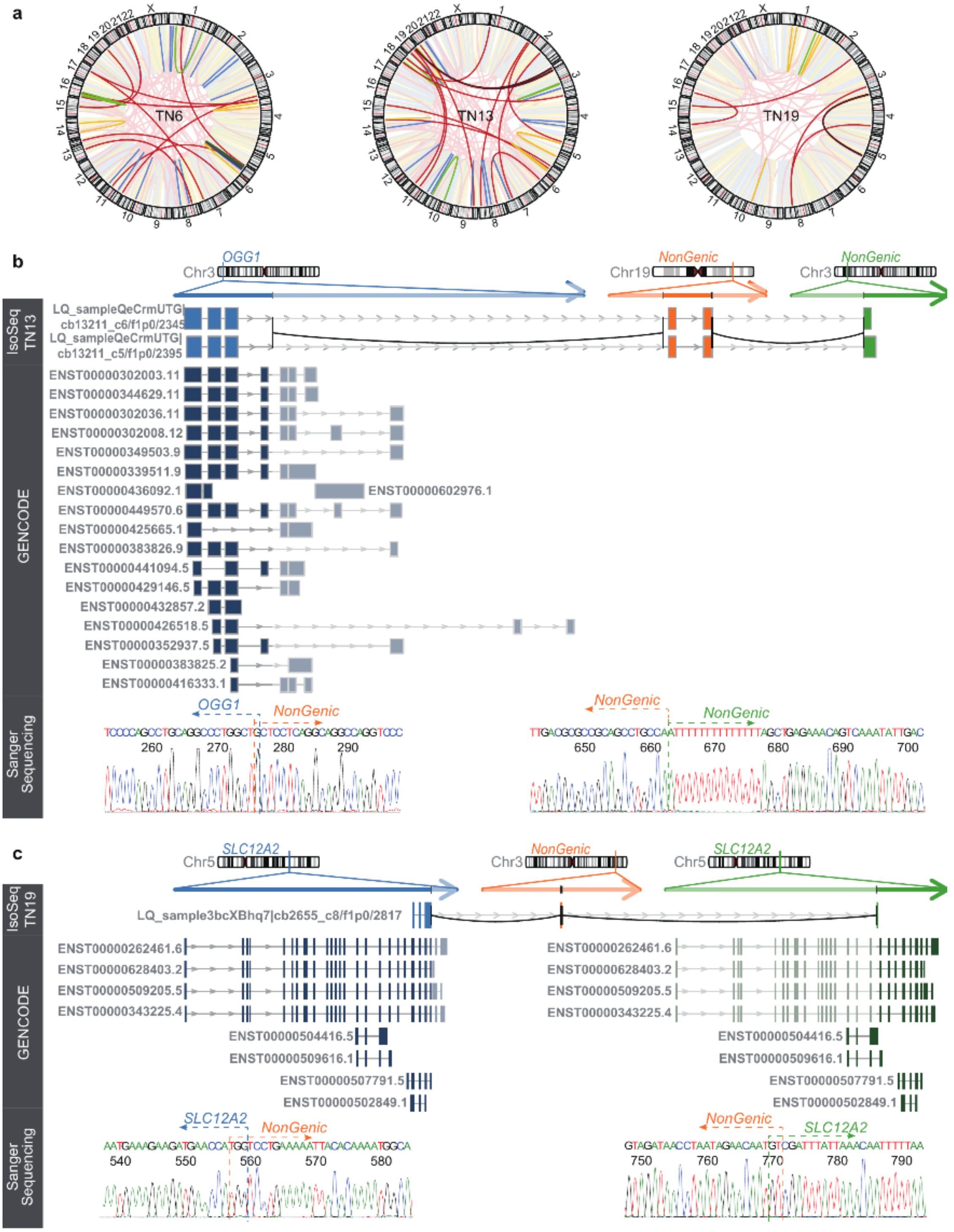

PCR-validated double-hop fusion transcripts other than Fig. 7. a, Circos plots of structural variants and fusion transcripts. Plots are shown for samples with the double-

hop fusions that were confirmed by Sanger sequencing of PCR amplicons. Colored lines and shaded lines represent structural variants (SVs) with or without corresponding fusion transcripts, respectively. Colors correspond to SV types: yellow, deletion; green, inversion; blue, tandem duplication; and red, translocation. Colors are darkened for nested SV with fusion transcripts. **b** and **c**, Structure of double-hop fusion transcripts from *OGG1–NonGenic–NonGenic* (**b**) and *SLC12A2–NonGenic–SLC12A2* (**c**). Genomic axes represent three original genomic regions. Below them are chimeric IsoSeq cluster reads. Curves correspond to SVs detected using whole-genome sequencing data. The category "GENCODE" shows annotated transcripts. Outside regions of SVs are shaded. To ensure visibility, exon–intron structures do not necessarily reflect accurate length.

Supplementary Figure 17

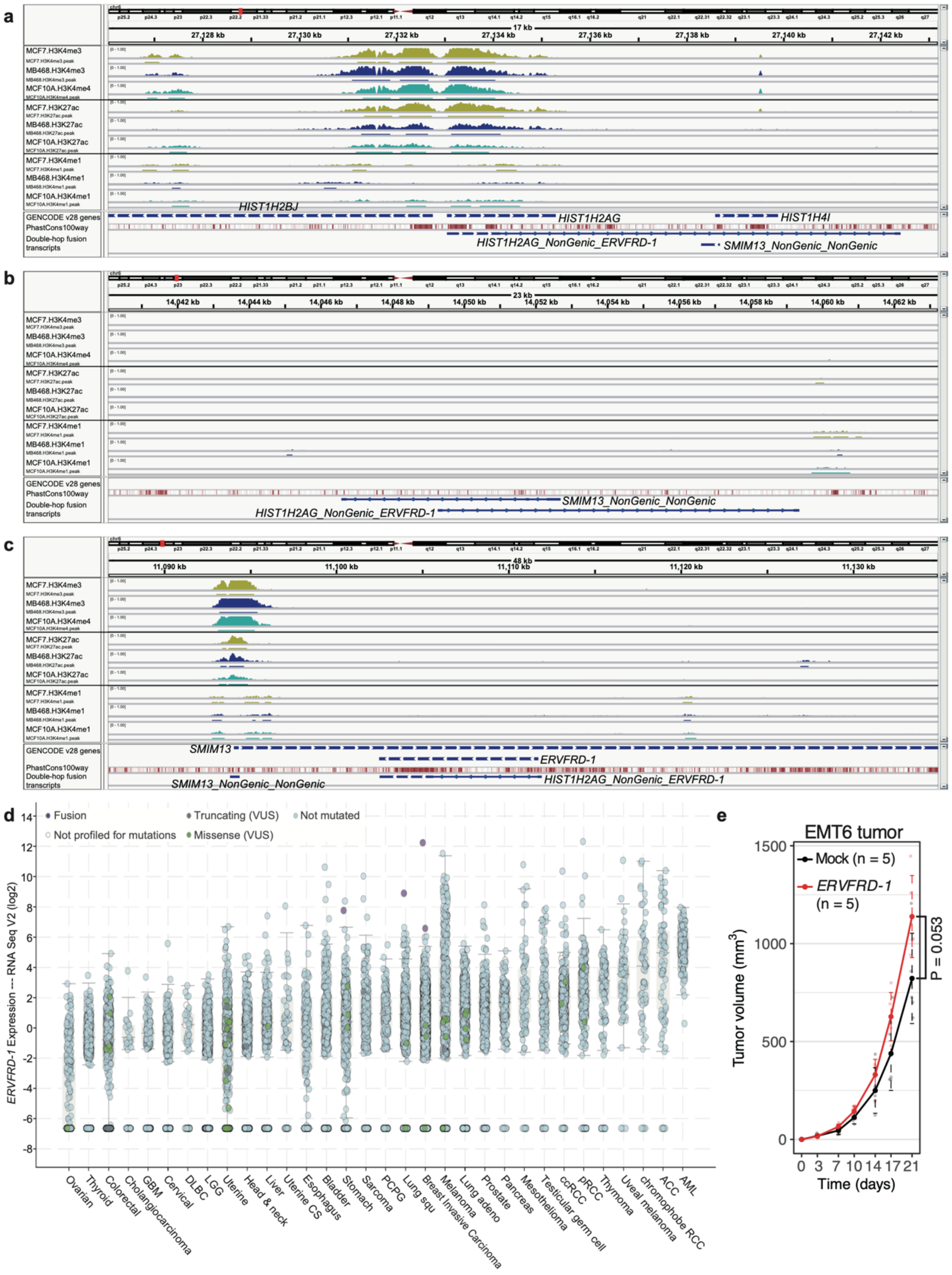

Supporting evidence for *HIST1H2AG–NonGenic–ERVFRD-1.* **a–c**, Chromatin modifications related to *HIST1H2AG–NonGenic–ERVFRD-1*. Integrative Genomics Viewer snapshots show the three regions where *HIST1H2AG* (**a**), *NonGenic* (**b**), and *ERVFRD-1* (**c**) fragments of the bridged fusion transcript *HIST1H2AG–NonGenic–ERVFRD-1* were located. **d**, *ERVFRD-1* in TCGA samples. Expression of *ERVFRD-1* gene across cancer types, colored according to somatic mutations and fusions. **e**, Growth of EMT6 tumor cells expressing *ERVFRD-1* in BALB/c mice. Error bars represent standard error.

Supplementary References

1. Soneson, C., Matthes, K. L., Nowicka, M., Law, C. W. & Robinson, M. D. Isoform prefiltering improves performance of count-based methods for analysis of differential transcript usage. *Genome Biology* **17**, (2016).

2. Gordon, S. P. *et al.* Widespread polycistronic transcripts in fungi revealed by single-molecule mRNA sequencing. *PloS one* **10**, e0132628 (2015).

3. Tang, A. D. *et al.* Full-length transcript characterization of sf3b1 mutation in chronic lymphocytic leukemia reveals downregulation of retained introns. *Nature communications* **11**, 1438 (2020).

4. Roller, M. *et al.* LINE retrotransposons characterize mammalian tissue-specific and evolutionarily dynamic regulatory regions. *Genome biology* **22**, 62 (2021).

5. Koch, L. Capturing transposases for new proteins. *Nature reviews. Genetics* **22**, 266–267 (2021).

6. Smit, A., Hubley, R. & Green, P. RepeatMasker open-4.0.

7. Li, H. Minimap2: pairwise alignment for nucleotide sequences. *Bioinformatics* (2018) doi:10.1093/bioinformatics/bty191.

8. Boratyn, G. M., Thierry-Mieg, J., Thierry-Mieg, D., Busby, B. & Madden, T. L. Magic-blast, an accurate rna-seq aligner for long and short reads. *BMC bioinformatics* **20**, 405 (2019).

9. Altschul, S. F. *et al.* Gapped blast and psi-blast: A new generation of protein database search programs. *Nucleic acids research* **25**, 3389–402 (1997).

10. Jones, P. *et al.* InterProScan 5: Genome-scale protein function classification. *Bioinformatics* **30**, 1236–40 (2014).

11. Götz, S. *et al.* High-throughput functional annotation and data mining with the blast2go suite. *Nucleic acids research* **36**, 3420–35 (2008).

12. Zhou, H., Yang, Y. & Shen, H.-B. Hum-mPLoc 3.0: Prediction enhancement of human protein subcellular localization through modeling the hidden correlations of gene ontology and functional domain features. *Bioinformatics (Oxford, England)* **33**, 843–853 (2017).

13. Wang, D. *et al.* DM3Loc: Multi-label mRNA subcellular localization prediction and analysis based on multi-head self-attention mechanism. *Nucleic acids research* **49**, e46 (2021).
