# Supplementary material for: Transcript-targeted analysis reveals isoform alterations and double-hop fusions in breast cancer
Source: Commun Biol. 2021 Nov 22;4:1320. doi: 10.1038/s42003-021-02833-4 (PMC8608905; doi:10.1038/s42003-021-02833-4)
Supplement: Supplementary file 2 — Description of Additional Supplementary Files [file 42003_2021_2833_MOESM2_ESM.pdf]

Description of Additional Supplementary Files

File name: Supplementary Data 1

Description: The number of reads and isoforms in each breast cancer clinical specimen.

File name: Supplementary Data 2

Description: Isoforms with splice sites created by somatic mutations.

File name: Supplementary Data 3

Description: Differentially expressed genes by the TNS3 short isoform or the canonical TNS3 isoform.

File name: Supplementary Data 4

Description: The association of the TNS3 short form with prognosis in TCGA.

File name: Supplementary Data 5

Description: Genomic positions of the double-hop fusions.
