# Supplementary material for: Transcript-targeted analysis reveals isoform alterations and double-hop fusions in breast cancer
Source: Commun Biol. 2021 Nov 22;4:1320. doi: 10.1038/s42003-021-02833-4 (PMC8608905; doi:10.1038/s42003-021-02833-4)
Supplement: Supplementary file 8 — Reporting Summary [file 42003_2021_2833_MOESM8_ESM.pdf]

nature research

Corresponding author(s): Masahito Kawazu

Last updated by author(s): Oct 27, 2021

Reporting Summary

Nature Research wishes to improve the reproducibility of the work that we publish. This form provides structure for consistency and transparency in reporting. For further information on Nature Research policies, see our Editorial Policies and the Editorial Policy Checklist.

Statistics

For all statistical analyses, confirm that the following items are present in the figure legend, table legend, main text, or Methods section.

n/a	Confirmed	
[ ]	[x]	The exact sample size (*n*) for each experimental group/condition, given as a discrete number and unit of measurement
[ ]	[x]	A statement on whether measurements were taken from distinct samples or whether the same sample was measured repeatedly
[ ]	[x]	The statistical test(s) used AND whether they are one- or two-sided<br>*Only common tests should be described solely by name; describe more complex techniques in the Methods section.*
[ ]	[x]	A description of all covariates tested
[ ]	[x]	A description of any assumptions or corrections, such as tests of normality and adjustment for multiple comparisons
[ ]	[x]	A full description of the statistical parameters including central tendency (e.g. means) or other basic estimates (e.g. regression coefficient) AND variation (e.g. standard deviation) or associated estimates of uncertainty (e.g. confidence intervals)
[ ]	[x]	For null hypothesis testing, the test statistic (e.g. *F*, *t*, *r*) with confidence intervals, effect sizes, degrees of freedom and *P* value noted<br>*Give P values as exact values whenever suitable.*
[x]	[ ]	For Bayesian analysis, information on the choice of priors and Markov chain Monte Carlo settings
[x]	[ ]	For hierarchical and complex designs, identification of the appropriate level for tests and full reporting of outcomes
[ ]	[x]	Estimates of effect sizes (e.g. Cohen's *d*, Pearson's *r*), indicating how they were calculated

Our web collection on statistics for biologists contains articles on many of the points above.

Software and code

Policy information about availability of computer code

Data collection	Whole genome sequencing reads were aligned to reference hg38 and somatic mutations and structural variants were called with Genomon (v2.6.0). RNAseq reads were mapped to reference hg38 and expression data was calculated in the format of transcript par million by 2 ways, quasi-mapping-based mode of Salmon (v0.12.0), and STAR (v2.5.2a) and RSEM (v1.3.1). Long-read transcript sequencing reads were summarized into consensus cluster sequences by IsoSeq2 pipeline using SMRTlink (v5.1.0.26412), underwent hybrid error correction with LoRDEC (v0.9), and subsequently mapped to reference hg38 by Minimap2 (v2.12-r847-dirty).
Data analysis	Multi-sample isoform assembly was conducted by MuSTA (https://github.com/shinichinamba/MuSTA). Isoform classification and filtering were performed by SQANTI (v1.2). Differential gene expression used R packages DESeq2 (v1.22.2) and tximport (v1.10.1). Differential transcript usage used R packages DEXseq (v1.28.3) and stageR (v1.4.0). SUPPA2 (v2.3) were used for alternative splicing.<br><br>Software used in the more detailed analyses were described in the manuscript: DRIMSeq (v1.10.1), HMMER (v3.1b2), psichomics (v1.8.2), and BLAST (v.2.9.0+).

For manuscripts utilizing custom algorithms or software that are central to the research but not yet described in published literature, software must be made available to editors and reviewers. We strongly encourage code deposition in a community repository (e.g. GitHub). See the Nature Research guidelines for submitting code & software for further information.

Data

Policy information about availability of data

All manuscripts must include a data availability statement. This statement should provide the following information, where applicable:

- Accession codes, unique identifiers, or web links for publicly available datasets
- A list of figures that have associated raw data
- A description of any restrictions on data availability

The raw sequencing data have been deposited in the Japanese Genotype-Phenotype Archive (https://www.ddbj.nig.ac.jp/jga/index-e.html) under accession number JGAS000095. The dataset ID for the whole genome sequence and RNA-seq is JGAD000095, and the dataset ID for Iso-seq is JGAD000457. Supplementary Data (the annotation of the MuSTA-derived transcriptome generated from 22 breast cancer specimens, the annotation of the novel predicted proteins, and the correlations of gene expression between intergenic genes and their neighbor genes) are available at Figshare (https://doi.org/10.6084/m9.figshare.16681219).

Field-specific reporting

Please select the one below that is the best fit for your research. If you are not sure, read the appropriate sections before making your selection.

☒ Life sciences ☐ Behavioural & social sciences ☐ Ecological, evolutionary & environmental sciences

For a reference copy of the document with all sections, see nature.com/documents/nr-reporting-summary-flat.pdf

Life sciences study design

All studies must disclose on these points even when the disclosure is negative.

Sample size	No statistical method was used to predetermine sample size. Sample size was determined by the availability of sequencing data.
Data exclusions	No data were excluded for analyses.
Replication	No experimental replication was performed for sequencing experiments.<br>For a simulation study with different novel in catalog (NIC) rates, we conducted three independent simulations with randomly generated sequences.<br>For in vivo and in vitro experiments, all attempts at replication were successful with biological replicates performed on separate cohorts of animals/cells.
Randomization	Since samples were assigned to groups based on their subtypes, randomization was not required.
Blinding	Not applicable. Our analyses (for example, differential transcript usage) were statistical tests that did not require blinding.

Reporting for specific materials, systems and methods

We require information from authors about some types of materials, experimental systems and methods used in many studies. Here, indicate whether each material, system or method listed is relevant to your study. If you are not sure if a list item applies to your research, read the appropriate section before selecting a response.

Materials & experimental systems

n/a	Involved in the study	
☒	☐	Antibodies
☐	☒	Eukaryotic cell lines
☒	☐	Palaeontology and archaeology
☐	☒	Animals and other organisms
☐	☒	Human research participants
☒	☐	Clinical data
☒	☐	Dual use research of concern

Methods

n/a	Involved in the study	
☒	☐	ChIP-seq
☒	☐	Flow cytometry
☒	☐	MRI-based neuroimaging

Eukaryotic cell lines

Policy information about cell lines

Cell line source(s)	Human embryonic kidney (HEK) 293T cells, the human mammary gland epithelial cell line MCF10A, and the murine mammary carcinoma cell line EMT6 were obtained from the American Type Culture Collection (ATCC). MC-38 (mouse colon carcinoma) cell line was obtained from Kerafast (Boston, MA).
Authentication	All cell lines were authenticated by the providers using karyotype, isoenzmes, and/or microsatellite profiling (short tandem repeat or simple sequence length polymorphism).
Mycoplasma contamination	Cultured cells were tested for mycoplasma contamination using a MycoAlert Mycoplasma Detection Kit (Lonza), according to the manufacturer's instructions.
Commonly misidentified lines (See ICLAC register)	The study did not involve commonly misidentified lines.

Animals and other organisms

Policy information about studies involving animals; ARRIVE guidelines recommended for reporting animal research

Laboratory animals	Female C57BL/6J, BALB/c, and BALB/c-nu/nu mice (5–7 week) were purchased from Charles River Laboratories Japan and used at 6–9 weeks of age.
Wild animals	The study did not involve wild animals.
Field-collected samples	The study did not involve samples collected from the field.
Ethics oversight	All mouse experiments were approved by the Animals Committee for Animal Experimentation of the National Cancer Center Research Institute. All experiments met the U.S. Public Health Service Policy on Humane Care and Use of Laboratory Animals.

Note that full information on the approval of the study protocol must also be provided in the manuscript.

Human research participants

Policy information about studies involving human research participants

Population characteristics	Anonymized tumor samples obtained from adult female were used in the study. Clinical information is not available except for anonymized pathologist diagnostic reports.
Recruitment	Patients with breast cancer treated at The Yamaguchi University and Mie University Hospital were recruited to this study. Tumors with sufficient tumor contents were selected.
Ethics oversight	The genomic analysis of primary tumor tissue samples was approved by the Ethics Committee of National Cancer Center Research Institute (#2015-202).

Note that full information on the approval of the study protocol must also be provided in the manuscript.
